# Stem Cells and Innate Immunity in Aquatic Invertebrates: Bridging Two Seemingly Disparate Disciplines for New Discoveries in Biology

**DOI:** 10.3389/fimmu.2021.688106

**Published:** 2021-06-30

**Authors:** Loriano Ballarin, Arzu Karahan, Alessandra Salvetti, Leonardo Rossi, Lucia Manni, Baruch Rinkevich, Amalia Rosner, Ayelet Voskoboynik, Benyamin Rosental, Laura Canesi, Chiara Anselmi, Annalisa Pinsino, Begüm Ece Tohumcu, Anita Jemec Kokalj, Andraž Dolar, Sara Novak, Michela Sugni, Ilaria Corsi, Damjana Drobne

**Affiliations:** ^1^ Department of Biology, University of Padua, Padua, Italy; ^2^ Middle East Technical University, Institute of Marine Sciences, Erdemli, Mersin, Turkey; ^3^ Department of Clinical and Experimental Medicine, Unit of Experimental Biology and Genetics, University of Pisa, Pisa, Italy; ^4^ Department of Biology, Israel Oceanographic and Limnological Research, National Institute of Oceanography, Haifa, Israel; ^5^ Institute for Stem Cell Biology and Regenerative Medicine, Stanford University School of Medicine, Stanford, CA, United States; ^6^ Department of Biology, Stanford University, Hopkins Marine Station, Pacific Grove, CA, United States; ^7^ Department of Biology, Chan Zuckerberg Biohub, San Francisco, CA, United States; ^8^ The Shraga Segal Department of Microbiology, Immunology and Genetics, Faculty of Health Sciences, Center for Regenerative Medicine and Stem Cells, Ben-Gurion University of the Negev, Beer-Sheva, Israel; ^9^ Department of Earth Environment and Life Sciences (DISTAV), University of Genoa, Genoa, Italy; ^10^ Institute for Biomedical Research and Innovation, National Research Council, Palermo, Italy; ^11^ Department of Biology, Biotechnical Faculty, University of Ljubljana, Ljubljana, Slovenia; ^12^ Department of Environmental Science and Policy, University of Milan, Milan, Italy; ^13^ Department of Physical, Earth and Environmental Sciences, University of Siena, Siena, Italy

**Keywords:** stem cells, immune cells, tissue regeneration, aquatic invertebrates, system biology, omics technology

## Abstract

The scopes related to the interplay between stem cells and the immune system are broad and range from the basic understanding of organism’s physiology and ecology to translational studies, further contributing to (eco)toxicology, biotechnology, and medicine as well as regulatory and ethical aspects. Stem cells originate immune cells through hematopoiesis, and the interplay between the two cell types is required in processes like regeneration. In addition, stem and immune cell anomalies directly affect the organism’s functions, its ability to cope with environmental changes and, indirectly, its role in ecosystem services. However, stem cells and immune cells continue to be considered parts of two branches of biological research with few interconnections between them. This review aims to bridge these two seemingly disparate disciplines towards much more integrative and transformative approaches with examples deriving mainly from aquatic invertebrates. We discuss the current understanding of cross-disciplinary collaborative and emerging issues, raising novel hypotheses and comments. We also discuss the problems and perspectives of the two disciplines and how to integrate their conceptual frameworks to address basic equations in biology in a new, innovative way.

**Graphical Abstract:**
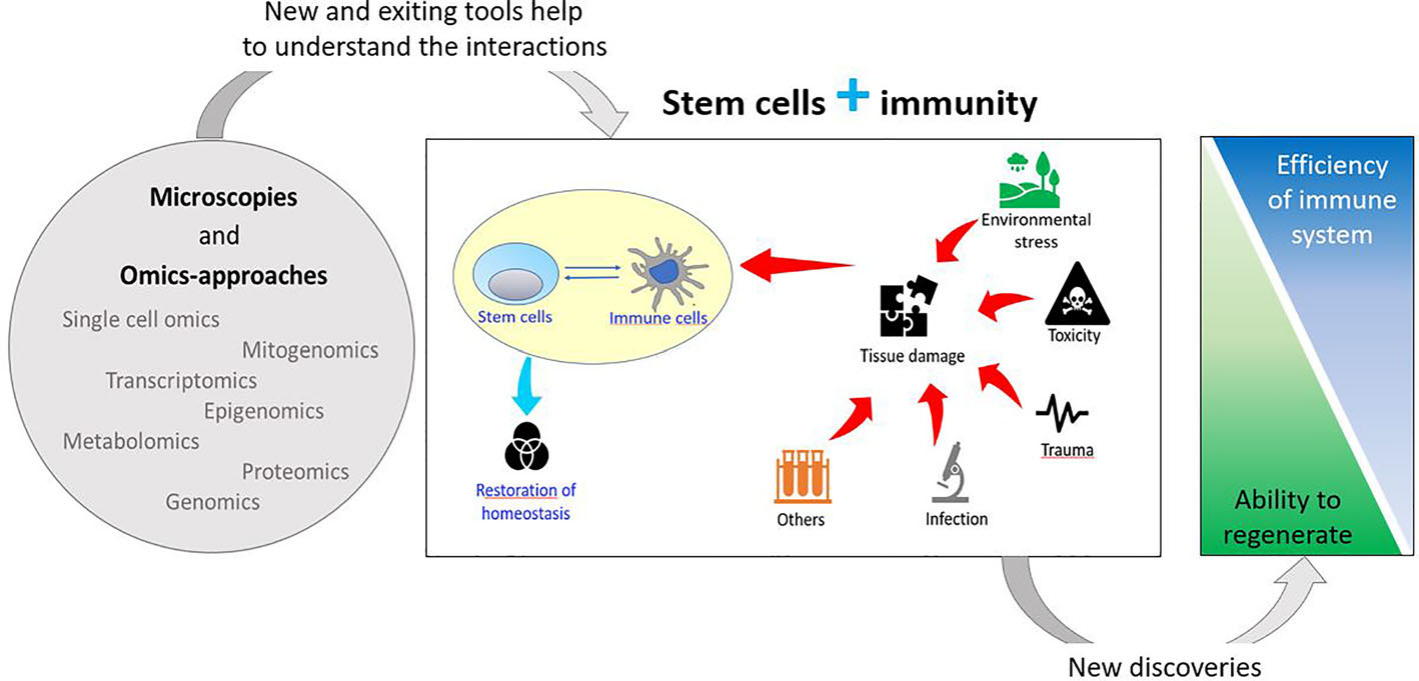


## Introduction

Stem cells are multipotent, self-renewing entities that generate one or more differentiated daughter cell types (1, 2). Conversely, immune cells are differentiated cells devoted to immunosurveillance and able to detect and respond to non-self stimuli. Stem and immune cells are thus commonly discussed as separate entities and considered to belong to disparate branches of biological disciplines with limited or even no interconnections. Yet, following Morin’s (3) view for any complex system as “composed of interweaving interactions characterized by complementary, antagonistic, concurrent relationships so that no one exists and can be understood in isolation from the whole”, it becomes natural to consider these two complex cellular networks, as not fully understood in isolation from each other. Stem and immune cells are essential for animal survival and fitness. Indeed, the two systems contribute to homeostasis by perceiving physiological changes in the systemic environment and, consequently, they generate differentiated cells or mount an immune response, respectively (4–6).

There are multiple examples in mammals, showing intermingled immune and stem cell functionalities (see below), whereas immunotoxicology and pharmaceutical research are additional scientific fields interfacing immunologists and stem cell biologists. As an example, human and murine colony-forming units-granulocyte/macrophage assays have been validated for assessing xenobiotic-induced myelotoxicity, and *in vitro* bone marrow stem cell assays are now commercially available and routinely used in pharmaceutical screening. The assessment of myelotoxicity provides a broad measure of the potential impacts of chemicals on the growth and development of immune cells, where all immune-related cells develop from pluripotent, hematopoietic stem cells residing in the bone marrow. This is not the situation in aquatic invertebrates, where stem cell-based bioassays have not yet been adequately explored as ecotoxicological tools (7).

Aquatic invertebrates represent the great majority of animal biodiversity and are an extremely promising group of organisms for stem cells and immune research. They can benefit from a more straightforward body organization, the abundance of adult stem cells spread throughout the body, and the lack of a complex immune network by lacking an adaptive immunity (8). Therefore, they represent ideal organisms to study the interplay between stem and immune cells.

Hematopoiesis is the driver for generating circulating immune cells able to face non-self and, in vertebrates, it has been the subject of intensive research by immunologists and stem cell biologists (9, 10). Conversely, in the majority of aquatic invertebrates, stem cell origin has not yet been adequately explored, while the immune response has been further confirmed as a relevant endpoint in ecological risk assessment of both legacy and emerging contaminants (8, 11).

Various stimulation experiments were carried out, and various techniques were used in aquatic invertebrates to study the hematopoietic tissue (HPT) in relation to the (circulating) immune cells (i.e., hemocytes, coelomocytes). The variations in haemocyte number are, most likely, mainly regulated by release from the HPT, perhaps complemented by storage and release of hemocytes in other sites (12). The majority of the cells in the HPT are able to proliferate, and proliferation can be increased significantly after the injection of saline or bacterial toxin like lipopolysaccharide (LPS) (13). There is a dynamic change in the HPT in response to LPS-induced blood loss, similar to that observed in response to an infection by bacteria (which contain LPS) or fungi (which contain laminarin) (14).

Although in many cases, the hematopoietic sites are still unknown, the mesothelium lining the coelomic spaces or the vasculature plays a vital role in the formation of the coelomocytes/hemocytes in spiralian coelomates as a source of hematopoietic stem cells (HSCs) that colonize specific lymphoid organs or lymph glands (15–17). Among invertebrate deuterostomes, in addition to the coelomic epithelium, the pharyngeal region acquires a role in hematopoiesis (18, 19).

In the present review, we will focus on the relationships between stem cells and the immune system in aquatic invertebrates, compared to vertebrates. We will highlight some selected examples stemming from the established collaboration within the MARISTEM COST Action (8). The introduction of modern techniques in biological research allows us to discuss new tools and frontiers in biological research aimed to study the links between stem and immune cells.

## Links Between Stem Cells and Immunity

The interplay between immune cells and stem cells is reciprocal (20), and the relationships between stem cells and immune responses have been particularly well studied in mammals. For example, the damage of the hair follicle bulge, the niche where skin stem cells reside, induces these cells to release stress signals that stimulate immune cells. Recruited immune cells, in turn, stimulate stem cell proliferation to restore niche integrity, thus depicting a scenario in which the two counterparts are finely intermingled in a reciprocal tuning (21). Mammalian mesenchymal stem cells can modulate immune responses by acting as antigen-presenting cells and immune suppressors (22, 23). In addition, HSCs discriminate non-self and respond to the infection through increased proliferation and differentiation of immune cells. The latter, during their differentiation, consequently to the recognition of non-self, acquire epigenetic modifications in immune genes that are transmitted to their descendants (24). Conversely, immune cells can regulate stem cells activity and differentiation. For instance, macrophages, far from being just professional phagocytes, can govern HSCs differentiation to blood cells and modulate stem cells differentiation in the mammary gland, epidermis and the intestinal crypts (25).

**Figure 1:**
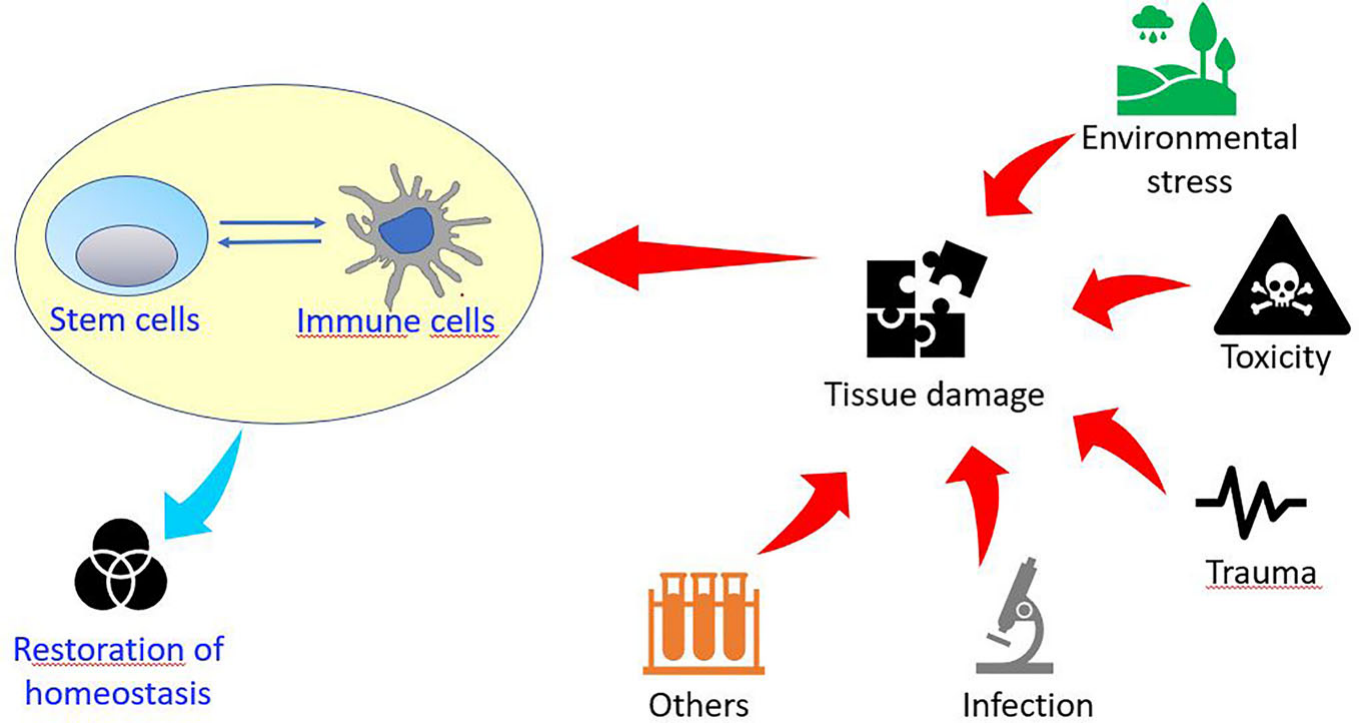
Schematic drawing of the relationships between stem and immune cells and their involvement in stress responses.

In addition, the immune system plays an important role in tissue/organ regeneration processes as immune cells contribute to the modulation of stem cell activation to facilitate regeneration. Regenerative organisms generally present limited pro-inflammatory responses, absence of scar formation and poor immune responses, suggesting an inverse correlation between regeneration capability and sophisticated adaptive immune system (20, 26, 27) (**Figure 1**).

Immune cells affect mammalian regeneration and tissue repair both positively and negatively (28). Different specific subtypes of macrophages (M) and T cells play opposite roles during wound healing, influencing the choice between regeneration and fibrotic repair. In accordance, regenerative capacity is impaired if anti-inflammatory processes initiate too early or if inflammation persists too long (20, 29). During scar healing, M (IFN-gamma) inflammatory macrophages activate T helper cells that release inflammatory cytokines inhibiting stem cells, while M (IL-4) pro-fibrotic macrophages induce extracellular matrix deposition. On the contrary, during scarless healing (M(IL-10), anti-inflammatory/anti-fibrotic macrophages activate subtypes of regulatory T cells that secrete anti-inflammatory cytokines, while the tissue-resident subtype γδτ cell may activate stem/progenitor cells by releasing growth factors (27).

Direct evidence of the interaction between the immune system and tissue regeneration has also been provided in the axolotl, where it has been demonstrated that macrophages, a crucial component of the innate immune system, infiltrate at the wound site and are essential for limb regeneration and their reduction causes regeneration failure and considerable fibrosis. Indeed, macrophage depletion induced deregulation of dedifferentiation factors, causing the disruption of the early regenerative blastema, formed by the accumulation of dedifferentiated multipotent cells (30). Moreover, myeloid cells are implicated in salamander lens regeneration (31) and the reduction of a specific subset of macrophages also influenced blastema formation and fin tail regeneration in zebrafish *Danio rerio* (32).

The cross-talk between immune cells, neural stem and progenitor cells and their progeny seems to determine both the efficacy of endogenous regenerative responses and the mechanism of action (33). Neurons do not efficiently regenerate in mammals and several studies suggest that the inflammatory response to injury impedes neurogenesis (34–36). In the zebrafish brain, which has the capacity to regenerate and replace neurogenic activity, recent work showed that inflammation is necessary and sufficient to initiate neurogenesis *via* progenitor cell activation. A similar phenomenon is also known in crustacean neurogenesis (37). Immune-mediated regeneration has also been discovered in skeletal muscle, a well-studied model for adult mammalian regeneration that employs activation of satellite cells, the resident progenitors of the muscle (38).

Unlike vertebrate immune systems, armored with the innate and the adaptive forms of immunity, invertebrates, which include more than two millions species and belong to more than 30 phyla, use only the ancient form of germline encoded innate immunity (39). Innate immunity in aquatic invertebrates, as in the vertebrates, relies on recognition elements (their nature not yet disclosed in most taxa), tightly associated with a wide range of effector mechanisms. While many prominent properties of innate immunity systems are shared by all multicellular organisms (40–46), the evolutionary origin remains poorly understood, as well as the roles of stem cells in immunological pathways of aquatic invertebrates (47, 48). Here, we present information on the relationships between stem and immune cells deriving from research on selected taxa of aquatic invertebrates.

### Planarians

**Figure 2:**
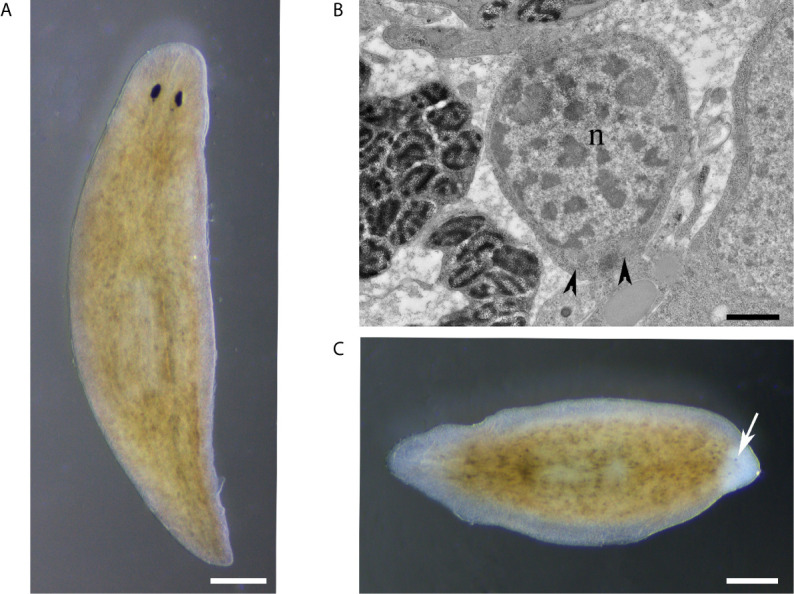
**(A)** Intact planaria (*Dugesia japonica*, asexual GI clone). **(B)** Electron micrograph of a neoblast, the planarian stem cell, with scanty undifferentiated cytoplasm containing free ribosomes and mitochondria. n, nucleus; arrowhead, mitochondria. **(C)** A tail fragment is regenerating the head, 3 days after amputation. Arrow indicates the unpigmented blastema containing the new differentiating eye cups. Scale bar: 100 μm in **(A, C)**, and 800 nm in **(B)**.

Planarians are highly regenerative organisms for which the potential role of the innate immune system during tissue regeneration is still largely unknown (20, 49). Their amazing regenerative capability resides in a heterogeneous population of adult stem cells, the neoblasts, which contains the sigma-neoblasts, a subpopulation of pluripotent stem cells (50). Following injury, neoblast proliferates and accumulates below the wound producing the blastema, where the lost structures differentiate (51, 52) (**Figure 2**).

Two peaks of apoptosis are required for proper tissue regeneration and reticular cells, a macrophage-like cell type, are recruited to clear the debris at the site of injury (53). Recently, the presence of subsets of cathepsin-positive cells was found. They have a dendritic-like shape and phagocytic activity and their specification is determined by the transcription factor foxF-1 (54).

Data support a connection between immune cell activity, stem cells, and regenerative mechanisms. Indeed, upon injury, planarians macrophages produce maresins, mediators of inflammation resolution, and when their synthesis is blocked by the lipoxygenase inhibitor baicalein, the tissue regeneration rate is reduced (55). The infection with bacteria (*Pseudomonas* sp.) has been linked to the inhibition of tissue regeneration through the activation of TAK1/MKK/p38 innate immunity signaling (56). Among the genes likely involved in innate immunity, some were found specifically activated following wounding in the presence of LPS (57). It has been demonstrated that planarians are resistant to infection by a broad range of pathogenic bacteria and the key resistant gene, MORN2, which is conserved in humans, has been identified (58), thus making planarians a useful model for the identification of innate resistance mechanisms (59). Given the availability of RNAi, transcriptomic and proteomic approaches in planarians, as well as the possibility of single-cell gene profiling by flow cytometry analysis and single cell-transplantation (60, 61), these invertebrates represent a model for understanding the mechanisms integrating immune responses to stem cells and tissue regeneration. As an example, identifying fundamental genes involved in reticular cell differentiation and their silencing by RNAi could select organisms with inhibited immune system which could serve to study regenerative performance.

### Bivalve Mollusks

Bivalves, the second largest group in Mollusca, are represented by several species, all of ecological importance in aquatic ecosystems, as well as of considerable economic value in estuarine and coastal areas (62). Their ability to face infection and their disease susceptibility have been deeply investigated in farmed species (i.e., as mussels, oysters, clams) and provided enormous progress in the characterization of their circulating cells, the hemocytes, including the immune cells and their cellular receptors and signal transduction pathways (63).

In the Mediterranean mussel *Mytilus galloprovincialis*, one of the most studied bivalve species, different subpopulations of hemocytes identified by flow cytometry have been functionally characterized with large and small granular hemocytes, representing phagocytes that can produce high levels of reactive oxygen species and nitric oxide upon activation (64). In contrast, small hyaline cells are not phagocytic and perform other defense mechanisms; they also include hemoblast-like cells that are suggested as possible stem cells in bivalves. The hemocyte half-life was determined to be between 22 and 28 days (65) in various bivalve species, thus suggesting the need for a continuous hemocyte replacement from stem/precursor cells. In the blue mussel *Mytilus edulis*, basal proliferation can account for up to 20% of the circulating cells indicating an essential process of hemocyte homeostasis (66). Although the identification of stem cells in mollusks is of physiological interest when considering the long-life span (years) and the extreme longevity of certain species (67), current knowledge on pluripotent cells in general, their location and their differentiation pathways, with special reference to immune cells, is still limited. Therefore, the origin of hemocytes remains an unanswered question in mollusks, including bivalves (16). In the Manila clam, *Tapes philippinarum*, newly formed hemocytes with stem cell-like features (according to cytochemical methods) can derive from the proliferation of undifferentiated circulating cells called hemoblasts (68). To date, the identification of a population of cells in the sub-epithelial connective tissue and vessels, resulting in positive for both SOX2, a stemness marker, and superoxide dismutase, a hemocyte marker, suggests that these cells are hemocyte progenitor cells and is still the only report on adult somatic progenitor cells in bivalves. It represents an essential first step in elucidating the hematopoietic process (65). The expression of SOX2 is significantly induced by LPS and Poly I:C, two well known activators of immune cells, in the freshwater species *Anodonta woodiana* (69), indicating an influence of immune cells on stem cells.

Overall, it is likely that adult bivalves do not produce hemocyte precursors or mature hemocytes from distinct hematopoietic organs, as it occurs in other molluscan taxa like gastropods (16). Multiple or ubiquitous sites of hematopoiesis may exist, comprising a system in which stem cells present in both tissues and circulation receive decisive signals from neighboring cells. The experimental challenge with bacteria, leading to changes in hemocyte subpopulations, was useful in identifying the expression of both proliferation and differentiation markers in circulating hemocytes (70–72).

Available information on bivalve hematopoiesis in larval stages has been recently reviewed (73). Based on multiple functional and molecular evidence for the presence of immune cells in the early developmental stages of various bivalve species, the author hypothesized that the development of the immune system is inextricably linked with the formation of the digestive system from the earlier trochophora, a key stage when the formation of the first ciliated digestive organs (such as the mouth, esophagus, and digestive mass) occurs. In this view, at the early stages of development, when pathogens can enter the body *via* feeding, immune cells would play a double role as digestive cells and immune cells. Developmentally, hemocytes are closely related to the epithelial cells lining the vascular system (endothelia) and the body cavity (mesothelia) and, in the absence of a circulatory system and HPT in early larvae, immune cells may be produced by precursor cells of the digestive system or associated mesothelial cells. Only later, when the circulatory system develops, hemocytes may be produced by tissues lining vessels and sub-epithelial cells (73).

### Crustaceans

Crustaceans represent a morphologically and physiologically diverse group of organisms, comprising between 40,000 and 60,000 species (74). They belong to an evolutionary very successful group of aquatic invertebrates characterized by life-long growth, able to enlarge their organs as adults and regenerate lost appendages.

Research on immune response in crustaceans has been largely driven by the need to understand diseases in aquaculture and to develop knowledge of environmental effects within a fishery context (75). Investigation of stem and progenitor cells in crustaceans has been primarily focused on the research of replacement of damaged and aged cells in the tissues to ensure homeostasis, immune responses and regeneration of lost appendages (76). This knowledge on how stem cells and immune cells communicate, sense damage and co-operate to help tissues to cope with stress is available only for some species of crustaceans.

Crustaceans have an open circulatory system, with a highly developed cardiovascular system. Like other invertebrates, their hemocytes have essential roles in immunity, performing recognition, cell–cell communication, lysis of foreign cells, melanisation, encapsulation, phagocytosis, and cytotoxicity. They are also involved in other physiological processes, such as the repair of the cuticle (77) or damaged skeletal muscles (78) and neurogenesis (79), where orchestrated action of immunity and stem cells is needed to maintain or restore homeostasis.

**Figure 3:**
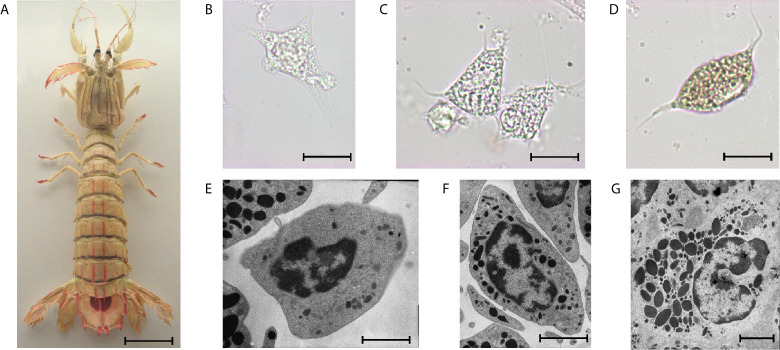
The mantis shrimp *Squilla mantis* **(A)**, an edible stomatopod crustacean, common along the European coasts, and its hemocytes. **(B–D)**: light microscopy; **(E–G)**: transmission electron microscopy; scale bar: 3 cm for **(A)**; 5 µm for **(B–D)**; 2 µm for **(E–G)**, **(B, E)**: hyaline cells; **(C, F)**: semigranulocytes; **(D, G)**: granulocytes.

The number of circulating hemocytes can vary significantly and depends on the physiological state (12). Crustaceans have three main types of hemocytes in their hemolymph, i.e., hyaline cells, semigranular cells, and granular cells (**Figure 3**), as well as immature progenitor cells. Indeed, crustacean hemocytes are produced and start their differentiation within the HPT, but the final differentiation into functional hemocytes occurs once the hemocytes are released into the circulation (80). Circulating hemocytes do not divide and aged cells must be continuously replaced by the release of new cells from hematopoietic organs (81). The variations in hemocyte number are, most likely, mainly regulated by the release of cells from the HPT, and perhaps complemented by storage/release of hemocytes in/from other sites. Although research on the role of hemocytes in crustaceans is rapidly progressing, the knowledge on HPT in different species is still limited.

In decapods, HPT comprises lobules with hemocyte precursors at different stages of differentiation (82). Each lobule is surrounded by connective tissue and contains stem cells, prohemocytes, and mature hemocytes, which are then released into the hemocoel (83). There are large variations in lobule morphology among species (13). Stem cells exclusively occupy the apical parts of the hematopoietic lobules and are closely attached to the extracellular envelope of the lobules. In the shrimp *Sicyonia ingentis*, the endothelial cells, capsular cells, and stromal cells, together with the extracellular matrix envelope hematopoietic lobules, and provide a niche-like microenvironment for the stem cells (82).

The HPT is not the only tissue harboring stem cells. The E-cells of the hepatopancreas, the neurogenic stem cells of the brain, the satellite cells of the heart and skeletal musculature are examples of adult stem cells (ASCs). Some of these cells are more or less continuously active, like the neurogenic and hematopoietic stem cells (84, 85), while others are periodically activated like the myogenic satellite cells (86) or the E-cells (87).

A cross-talk between immune cells and stem cells was identified by Benton et al. (88), who demonstrated that hemocytes are a source of adult-born neurons in crayfish and showed that the immune system is a key contributor to adult neurogenesis. Neurogenesis is an ongoing process in the brains of adult organisms. Noonin et al. (14) described the anterior proliferation organ in *Pacifastacus leniusculus*, located at the anterior part of the HPT, near the brain, as an organ regulating stem cell activity, demonstrating a physical link between the HPT and the brain. Benton et al. (88) also reported that manipulation of circulating hemocyte levels results in highly predictable changes in the number of neurogenic niche cells. These data show that the neurogenic niche is dynamically regulated by the immune system.

Crustaceans can partly regenerate their hepatopancreas, hearts and ovaries: here, similar ultrastructural features were observed in regenerating tissues of early adults and old specimens and included active stem cells (89). The immune cells have only recently emerged as key components and prominent effectors of stem cell behaviors to help stem cells in maintaining tissue integrity and driving regeneration (25). Common functions of the immune response in regeneration include scavenging cellular debris and activating progenitor cells as well as production of cytokines, and complement factors (90). In the hepatopancreas, hovering between cell proliferation and cell death (mainly apoptosis) plays an important role in the maintenance of tissue homeostasis. Hemocyte infiltration, in the form of an inflammatory-like reaction, was also observed in the hepatopancreas of *Palaemonetes argentinus* under stress conditions leading to increased desquamation of necrotic epithelium (91).

### Echinoderms

Echinoderms are very successful and ancient marine invertebrates, which appeared more than 500 years ago, before the Cambrian explosion (92). Many of them (e.g., sea urchins, sea cucumbers) play a central role in the safeguarding of certain marine ecosystem integrity and are constantly exposed to environmental and anthropogenic pressure, including predation, climate changes, pathogens, and pollutants (e.g., chemicals, nanomaterials, plastics) (93). This favors phenotypic plasticity both in the embryonic and adult life-cycle stages, thus conferring a notable ability to generate multiple phenotypic shapes and responses towards an always-changing world (94).

Echinoderm immune cells are the so-called coelomocytes, a heterogeneous population of cells freely circulating in all coelomic spaces, including the perivisceral coelomic cavities, the open water-vascular system, the perihemal system, but also in the main tissues and organs (95–97). A general consensus on echinoderm coelomocyte types is lacking, possibly due to the actual diversity of morphotypes among the different classes (93) but mostly to the diversity of techniques and protocols used to analyze them (98). For this reason, from two to up to twelve cytotypes are reported in the literature (97, 99–105). The most common types are phagocytes, red and white amoebocytes, and vibratile cells (93, 97). Gorshkov et al. (101) described mature and immature coelomocytes, the latter possibly representing a progenitor population of the former. Echinoderm immune cells carry out many functions, such as clot formation, phagocytosis, encapsulation, clearance, oxygen transport, inflammation and cell recruitment at the wound and regenerative sites (93). They are a big source of bioactive molecules secreted into the coelomic fluid to maintain homeostasis and intercellular crosstalk (106). Under homeostatic conditions, these cells work together to maintain physiological balance within the organism while, under perturbation, the stimulus may induce an energetically expensive reprogramming of the immune cells, resulting in either a reduced reactivity (tolerogenic immunological response) or an increased response (potentiation) (107, 108).

The exact origin of coelomocytes is still a matter of debate. The coelomic epithelium is the favored HPT, as proposed by the pioneering work of Bossche and Jangoux (109) and further supported by more recent studies (105, 110, 111). Other structures/organs have also been proposed such as Tiedmann’s body, the axial organ and more recently the pharynx (19, 112, 113). It must be stressed, however, that all these “alternative” structures are always more or less directly linked to coelomic spaces and, therefore, to coelomic epithelium. A recent study by Sharlaimova et al. (105, 114) provided ultrastructural evidence of the release of different cell types from the perivisceral coelomic epithelium, thus demonstrating a hematopoietic role for this tissue. According to their model, the free-swimming population of coelomocytes is physiologically replenished by the coelomic epithelium through both apical detachment of differentiated cells and small scarcely differentiated cells. The latter could be regarded as stem/progenitor cells of at least a subpopulation of coelomocytes (114). The presence, although rare, of these undifferentiated cells within the coelomic lining would be consistent with the proliferative activity often reported in this tissue in both physiological and regenerating conditions (19, 110, 115).

**Figure 4:**
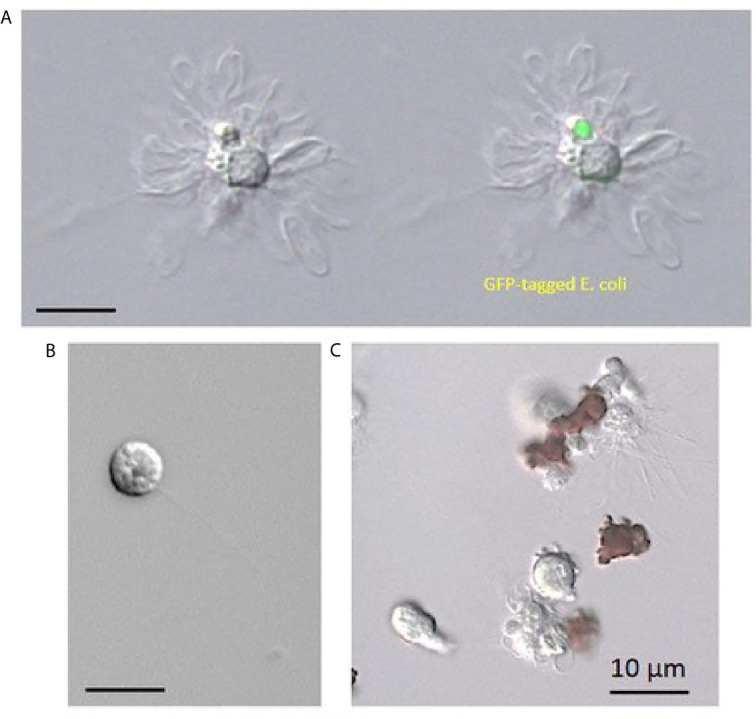
Immune cells from *Paracentrotus lividus* sea urchin collected as a total cell population in an anticoagulant solution containing EGTA. **(A)** Phagocytes, **(B)** vibratile cell, **(C)** red and white amoebocytes.

Some echinoderms possess extraordinary regenerative abilities (116, 117) in which immune cells play a fundamental role. Sea urchin diseases, consequent to lesions associated with body injuries or abrasions, induce loss of spines and other appendages and a bald patch, sometimes associated with a green mark probably due to a cellular infiltration into the affected area, which starts wound repair and drives tissue regeneration and recovery. Regardless of the origin of the wound (disease, autoamputation, or traumatic insult), immune cells are recruited first to create a clot (117) then to clear debris and pathogens as well as secrete numerous signaling molecules inducing appropriate cell proliferation and differentiation program essential for successful regeneration (20). Besides providing new circulating coelomocytes, the coelomic epithelium can also provide progenitor cells for the reconstruction of the regenerating tissue as reported in crinoids (116). This can occur thanks to a dedifferentiation process followed by epithelial-mesenchymal transition, allowing migration of scarcely differentiated cells in the underlying developing connective tissue (117–120). After arm amputation, trauma-stressed sea stars (*Asterias rubens*) show an increase both in the total number of the circulating coelomocytes and in the levels of Hsp70 stress protein within the cells. Besides, a protein known to be involved in coelomocyte adhesion (toposome) is massively expressed in the coelomic epithelium of trauma-stressed arms of *A. rubens* (121) (**Figure 4**).

### Ascidians

**Figure 5:**
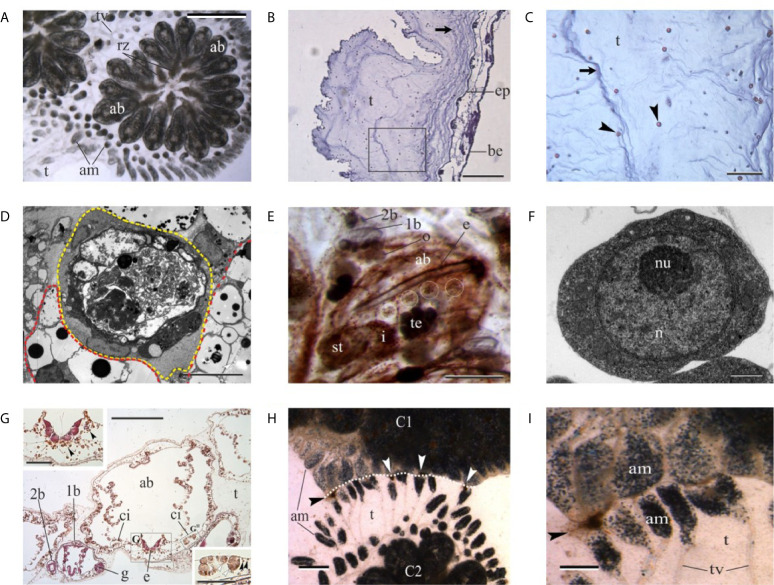
**(A)** Colony of the tunicate ascidian *Botryllus schlosseri*. The colony has concluded the change of generation and adult blastozooids (ab) coexist together with regressing zooids (rz). am, blood ampullae in tunic; t, tunic; tv, tunic vessel. Dorsal view. Scale bar: 2.2 mm. **(B, C)** Histological sections of the tunic of the solitary ascidian *Ciona robusta*. Square area in **(B)** is enlarged in **(C)** to show tunic fibers (arrows) and cells (arrowheads). Hematoxylin–eosin. be, branchial epithelium; ep, epidermis. Scale bar in B: 200 µm; in C: 50 µm. **(D)** Transmission electron microscopy of immunocytes in *B. schlosser*i. Yellow dotted line: large phagocyte with phagosomes of different size. Red dotted line: morula cells characterized by large vacuoles with an electrondense core. Scale bar: 10 µm. **(E)** Ventral view of an adult blastozooid (ab) of *B. schlosseri* with its primary bud (1b) bearing a secondary bud (2b). White circles label the cell islands in the left ventral body wall. Whole mount, ventral view; hematoxylin. o, oocyte in gonad rudiment in primary bud; i, intestine; st, stomach; te, testis. Scale bar: 500 µm. **(F)** Transmission electron microscopy of a candidate stem cells in *B. schlosser*i. n, nucleus; nu, nucleolus. Scale bar: 1 µm. **(G)** Transverse histological section of an adult blastozooid (ab) of *B. schlosseri* with its primary bud (1b). The latter bears a secondary bud (2b). The endostyle (e) niche and the cell island (ci) on the right are enlarged in upper left and bottom right insets, respectively. The contralateral cell island is contained by the black circle. Note in insets that small hemoblasts (black arrowheads) are recognizable in both the endostyle niche and in the cell island. In the latter, they are close to large phagocytes (white arrowheads). g, gonad rudimenti in primary bud; t, tunic. Hematoxylin–eosin. Scale bar in G: 200 µm; in G^I^-G^II^: 50 µm. **(H, I)** Regression between two incompatible colonies (C1 and C2) of *B. schlosseri*. Necrotic areas (arrowheads) are recognizable between the contacting tunics (white dotted line). am, blood ampullare; t, tunic; tv, tunic vessels. Scale bar in H: 1.2 mm; in I: 0.47 mm.

Ascidians are members of the subphylum Tunicata, the sister group of vertebrates (122). Their defense from environmental assaults relies mainly on the tunic and the circulating immune cells in the adults, and, likely, on the test cells in early embryonic stages (**Figure 5**).

The tunic is the external layer embedding the ascidian body (**Figures 5A–C**). It includes a gelatinous matrix rich in fibers (collagen, tunicin). It is always speckled with several types of free cells, some of which are permanently located within the tunic, while other cell types perambulate between the tunic and the body wall (123, 124). In some species, the tunic contains hemolympathic vessels (125). Among the various types of cells found within the tunic, there are cells functioning in innate immunity and allorecognition (phagocytes), chemical defense (bladder cells), light protection (pigmentary cells), photosymbiosis (phycocytes), tunic contraction (myocytes); for some of the tunic cells (e.g., granulocytes, amoebocytes), the functions are unknown (123). The tunic is secreted by the epidermal cells and tunic cells derive from both the epidermis and the hemocytes that enter the tunic in response to infections (123, 125, 126).

Circulating immune cells of ascidians are represented by phagocytes and cytotoxic morula cells (MCs), the latter containing the enzyme phenoloxidase inside their granules (127) (**Figure 5D**). Both the immunocyte types assure the organis’m defense against non-self and, in colonial species, trigger the inflammatory response consequent to allorecognition (128).

In *Botryllus schlosseri*, a model species for the study of immunobiology (**Figure 5A**), allorecognition and asexual reproduction, including whole-body regeneration (119, 129–131), colonies increase their size through periodical cycles of asexual reproduction requiring the involvement of stem cells. Unlike most species, where the body is long-lived and maintained by cellular replacement, *B. schlosseri* regenerates new colonial units (buds) on a biweekly basis from stem cells that remain for life, replacing the previous generation’s zooids and, partly, the circulatory cells (129, 132–137), Circulating immune cells influence the process as they assure the cross-talk between blastogenic generations at the generation change (takeover) (**Figure 5D**): cytotoxic MCs increase the transcription of the complement factor 3 (C3) and stimulate phagocytosis, required for the removal of apoptotic cells in tissues of old zooids (138, 139). Phagocytes are also required for the completion of the takeover and the formation of new buds as the prevention of phagocytosis blocks the process (140).

By limited dilution transplantation of cells that express the high enzymatic activity of aldehyde dehydrogenase, a stemness marker, and from a set of serial engraftment assays, Laird et al. (141) revealed that multipotent stem cells are responsible for stable long-term chimerism and budding in *B. schlosseri* colonies. A major adult somatic stem cell niche was further identified in the anterior ventral side of the endostyle (18), the long glandular groove extending medially at the ventral face of the zooid pharynx (**Figures 5E–G**). It is immersed in the hemolymphatic flow through the large subendostylar sinus and other sinuses (142). Cells that contribute to gonad formation were identified in cell islands along the endostyle (143). These stem cells and stem cell niches were identified by direct visualization of cells that exhibit fundamental and important aspects of mammalian stem cell biology, including self-renewal, homing into developing buds, expansion, differentiation, and multilineage potential (18, 141, 143). The involvement of circulating stem cells in the formation of new buds is also supported by the case of vascular budding or whole-body regeneration, where new buds can originate from the aggregation, inside the tunic vessels, of undifferentiated circulating cells even in the absence of adult zooids (144–152).

**Figure 6:**
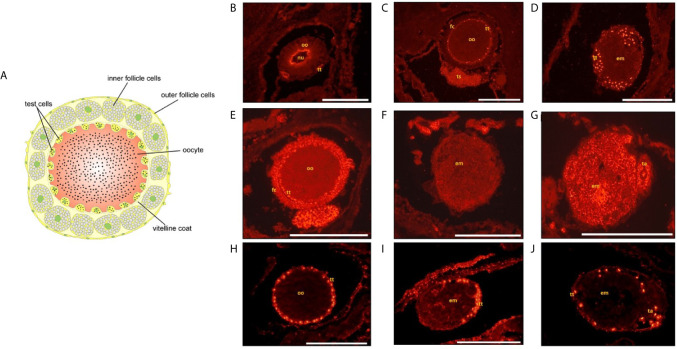
**(A)** Scheme of *Ciona robusta* oocyte showing the envelopes surrounding it. Test cells are located under the vitelline coat. Right: immunohistochemical analysis of *Botryllus schlosseri* oocytes **(B**, **C**, **E**, **H)** and embryos **(D**, **F**, **G**, **I**, **J)** with Vasa **(B–D),** PI10 **(E–G)** and IAP antibodies **(H–J)**. Bar=250 µm.

Ascidian eggs are enveloped by outer and inner follicle cells, the vitelline coat and the test cells (125). In *B. schlosseri*, all these cells share common surface antigens with hemocytes and tunic cells (153). Test cells constitute an ascidian-specific cell envelope encased in oocyte depressions beneath the other egg envelopes. During development, test cells adhere to the tunic surface and are discharged at hatching (154). Test cells are of maternal origin and are first detected during early oogenesis (155). They express markers that can be associated with immune defense but also with the stem lineages: (i) DDX1 (156) known to function as sensors of viral and bacterial RNAs and in inhibition of virus replication (157, 158); (ii) a IAP orthologue [IAP28 (159); **Figure 6**] that was correlated with cell proliferation control (160) and activation of caspases leading to apoptosis, depending on its subcellular location; (iii) Vasa and DDX3 (PL10), RNA helicases highly associated with soma/germ stem cells and germ lineages [(156, 161); **Figure 6**]. PL10 is also a modulator of the NF-κB pathway, known to be involved in responses to stress and viral/bacterial infections.

### Allorecognition in Aquatic Invertebrates: Stem Cell-Related Immunity

The term allorecognition is generally defined as the capability of intraspecific recognition of non-self. Here, we will consider the animal immune-related phenomena of allograft rejection/acceptance and colony specificity that usually implies the triggering of an inflammatory reaction leading to the killing of the alien tissues.

Allorecognition is central to the understanding of innate immunity in vertebrates (39), and is one of the most highlighted features of aquatic invertebrates. It reflects the facility for self and non-self discrimination between conspecifics, a phenomenon that evolved over 600 million years ago, in concert with the development of multicellularity (162, 163), and reveals, with the few available examples on the formal genetics of allorecognition in marine invertebrates, levels of polymorphism at allorecognition loci that exceed typical levels associated with other polymorphic loci (164). One such example is the colonial tunicate *B. schlosseri*, where populations from several sites, worldwide, revealed 80 to 300 allorecognition alleles at the fusibility locus/site (165–168). Indeed, while no homology has been established between marine invertebrates and vertebrates allorecognition genes, and not even among those of different invertebrate species (47, 169–171), a wide range of marine invertebrates, including sponges, cnidarians, bryozoans, and ascidians, reveal allorecognition competencies that represent high specificity and accuracy (44, 172, 173), also reflecting highly conserved effector mechanisms for innate immunity in the animal kingdom (40, 42–44, 46). The discovery of specific and complex non-transitive hierarchies in marine invertebrates (174–176) is dictated by a variety of effector mechanisms that are tailored to recognize different conspecifics (177).

Allorecognition in aquatic invertebrates has been probably developed for purposes such as the regulation of gametic compatibility (164, 178), for the discriminatory machinery of within-organism conflicts (179), as a platform to purge nascent selfish cells (39), as a partisan tool following the completion of each major evolutionary transmission (180) and for protection against predatory cell lineages of the same species, part of the costs of fusion with non-self, where stem cell lineages play a major role (47, 181–184). It is manifested by specificity, immune priming, allogeneic maturation and natural chimerism (176, 185–188), many of them managed by stem cells. These allorecognition phenomena exhibit suites of effector mechanisms (specific and non-specific) that are based on extreme allotypic diversity, commonly emerged in wide groups of marine invertebrates, and are recurrently seen in nature in the wake of tissue contacts among conspecifics. The genetic blueprints of allorecognition in marine invertebrates were elucidated in just two model species, the hydrozoan *Hydractinia symbiolongicarpus* (189, 190) and the colonial ascidian *B. schlosseri* (171). Yet, transcriptomic profiling for other allorecognition responses also exists for other marine invertebrates, like sponges (191) and corals (44).

#### Ascidian Allorecognition as an Immune Phenomenon

In solitary ascidians, allorecognition is observed morphologically as fusion/rejection reactions of allografts (192, 193) or *in vitro* as “contact reaction” responses among co-cultured hemocytes (194–196). Both allorejection and contact reactions trigger inflammatory responses with a decisive role of cytotoxic hemocytes, known as MCs (197) that underwent degranulation and induced cytotoxicity (99). In *Styela plicata*, during the allograft rejection, there is significant recruitment of MCs within the allograft bed and the neighbor tissues within days of transplantation, followed by a slower increase of circulating undifferentiated cells, commonly known as lymphocyte-like cells or hemoblasts, in the tunic surrounding the graft. This response reaches a peak around day 30 from the transplantation (99, 193). Since MC-induced inflammation leads to cell death (see below), the hemoblast increase suggests the activation of hematopoietic activity with the release in the circulation of new cells, in order to replace the effete cells. This idea is further corroborated by the observation that, in *Styela clava*, the injection in the tunic of allogeneic hemocytes induces the proliferation of hemoblasts with a peak at 5 days post-injection (198).

**Figure 7:**
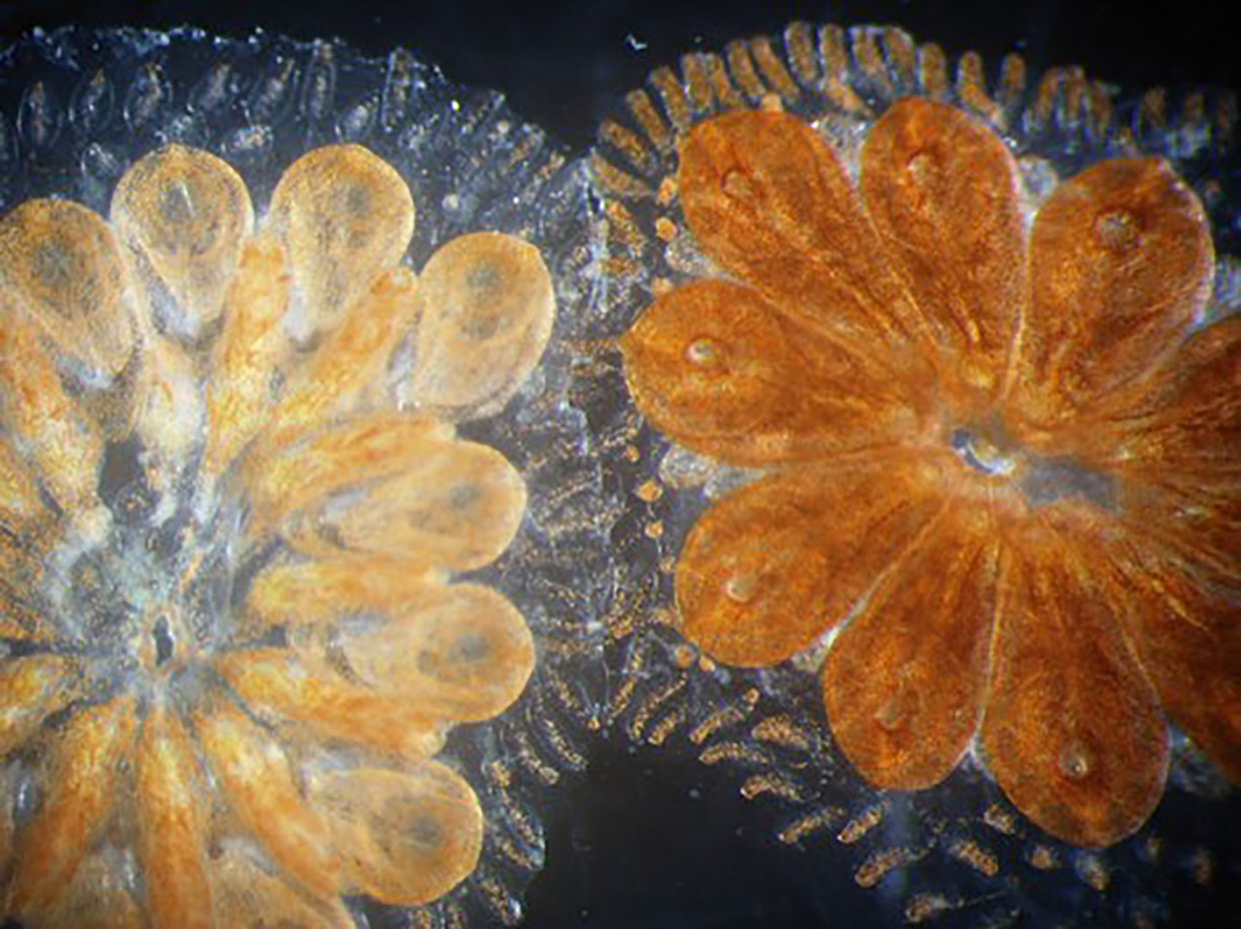
*Botryllus* chimera. Two genetically compatible colonies fused into a single chimeric colony, sharing the tunic and the circulatory system, after their contact.

In colonial ascidians, allorecognition manifests itself as colony specificity occurring whenever colonies contact each other. This leads to either a fusion between genetically compatible colonies, with the formation of a chimeric colony (**Figure 7**), or a rejection between incompatible colonies, with the appearance of a region of dead tissue along the contact border. In *Didemnum vexillum* allogeneic partners may, initially, form chimeric entities. Subsequently, zooids of differing genotypes coordinatively retreat away from the fusion zones and within a few days, they detached leading to the formation of zooid-depauperate tunic zones (199). The genetic control of fusion/rejection was particularly studied in the colonial species of the genus *Botryllus*. As previously reported, fusibility is controlled by a highly polymorphic fusibility/histocompatibility locus and fusion always occurs when contacting colonies share at least one allele at this locus (200–202). Fused colonies share both the tunic and the circulatory system. Without a common allele on the fusibility locus, an inflammatory (rejection) reaction follows the initial limited fusion of the tunics that allows the diffusion of soluble factors from one colony to the alien one. The outcomes of the rejection reaction are a series of melanic, necrotic spots along the contact border known as points of rejection (128, 203–206). At the cellular level, upon the recognition of the alien factor(s), MCs exit the colonial vasculature and enter the tunic at the contact points where they degranulate. The released materials, mainly the enzyme phenoloxidase and its polyphenolic substrata, induce a local cytotoxicity (128, 205–209). A “contact reaction”, similar to the one observed in solitary ascidians, leading to the release of fluorescent material from MCs and their ultimate death, together with that of neighboring cells, can also be observed when hemocytes from incompatible colonies are mixed *in vitro* (210). This implies that the dead cells must be replaced by new cells entering the circulation from hematopoietic sites. Indeed, experiments carried out in the Japanese species *Botryllus primigenus* indicate that X-ray irradiation can alter the intensity of the rejection reaction, likely affecting stem cells, commonly known as X-ray-sensitive cells (211). As a second step in allorecognition, the progressive resorption of one compatible partner frequently occurs (203, 212–214). The resorption of the zooids can be mimicked by the injection in the colonial vasculature of MCs from a fusible partner (215).

#### Ascidian Allorecognition and Stem Cell Lineage Competition

When colonies of *B. schlosseri* with opposite genotypes (AAbb and aaBB, respectively), relative to two Mendelian loci controlling zooid pigmentation, were separated after a short (few days) period of fusion, and crossed with the double recessive aabb, in addition to the expected offspring phenotypes (Ab and aB), a certain number of colonies with the aB and Ab phenotypes were obtained from the AAbb x aabb and aaBB x aabb crosses, respectively. This is indicative of the persistence of germ stem cells from the previously fused colony competing with the host stem cells to produce gametes (216). As, during the cyclical (weekly) generation replacement (blastogenesis) of *Botryllus* colonies (129), stem cells leave their temporary niches and, through the circulation, move to new niches in the growing buds (18, 143), it was possible to record the production of heterochtonous offspring for 15 blastogenetic generations and, in few cases, the entire offspring was heterochtonous, indicative of the complete germline parasitism of the host gonad by the alien stem cells (216).

Somatic parasitism can also be demonstrated when compatible colonies sharing one allele at the Fu/Hc locus (e.g., with AC and AD genotypes) were fused for at least four days before their separation. When AC colonies previously fused with AD colonies and AD colonies previously fused with AC colonies were challenged with BC and BD colonies, respectively, it was possible to observe an alteration of fusibility as colonies alternated fusibility and rejection with BC and BD colonies. This alteration, in few cases, was observed up to 2 years from the initial fusion and is indicative of the persistence of a renewing population of cells from the alien colony responsible for the observed effect. This is supported by the observation that the electrophoretic patterns of the enzyme phosphoglucoisomerase and amplified fragment length polymorphism in the AC colonies previously fused with AD colonies combine the pattern of AC and AD colonies (217–219).

In addition, as reported before, both somatic and germ cell parasitism can be obtained through the injection, in the circulation of compatible colonies, of hemocytes selected based on their high aldehyde dehydrogenase activity, a marker of stem cells in mammals (141).

Even in the case of colony resorption, the somatic and germline of the resorbed colony is not lost. Germ stem cells can invade the host gonads and give rise to germ cell parasitism with the production of heterochtonous offspring (203, 217, 219–221); somatic stem cells also persist in previously fused colonies, as demonstrated using microsatellites as molecular markers (220, 221). In addition, a chimera can express one or another genotype according to changes in seawater temperature (219), indicative of the capability of a colony to modulate its stem cell reservoir when the environment changes.

Although there are no direct data on the proliferation of circulating hemoblasts following allorejection (but see (184) for hemocyte xenotransplantations) or alloresorption, in both cases a MC-driven inflammatory event is activated leading to the ultimate death of these cells upon their degranulation (215). This suggests that, similarly to allograft rejection and contact reaction in solitary ascidians, new hemocytes should enter the circulation as a product of hematopoiesis activation. The relationships between inflammation and hematopoiesis, still unclear, deserves further investigation in the next future.

### Stem Cells-Immune Cells Cross-Talk in Stress and Toxicity

Immune and stem cells of invertebrate species have already been recognized as valuable models in ecotoxicology for both *in vitro* and *in vivo* studies. However, rarely the behavior of both cell type and their cross-talk in preserving and restoring homeostasis have been used as ecotoxicological endpoints (7, 11).

Recently, the potential application of aquatic invertebrate ASCs in ecotoxicology has been underlined in a very comprehensive review (7). The authors point out that, due to their lower genetic complexity, aquatic invertebrate ASCs represent a valuable and reliable tool for understanding fundamental biological processes, mode of action of pollutants and mechanisms of epigenetic toxicity. It was suggested that ASCs from selected invertebrate species having a key role in aquatic ecosystems could be harnessed in ecotoxicological testing.

**Figure 8:**
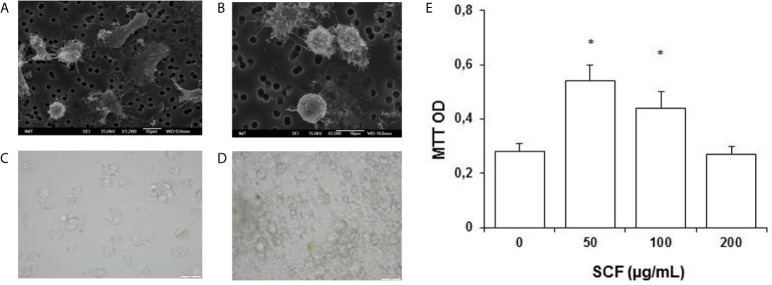
*Mytilus galloprovincialis*. **(A, B)** Scanning electron microscopy (SEM) image of freshly isolated cells (courtesy of Dr. Manon Auguste). **(C, D)** Light microscopy of control hemocytes **(C)** and hemocytes exposed to human Stem Cell Growth Factor (SCF) (24 h, 50 µg/mL, in the presence of 5 µg/mL Con-A **(D)**. **(E)** Effects of SCF on hemocyte proliferation evaluated by the MTT assay. *P < 0.05 Mann-Whitney U test.

However, up to now, the majority of ecotoxicological studies largely involved the use of immune cells instead of stem cells (11, 97, 222, 223). Few contributions investigating immune cells responses towards pollutants include aspects of hematopoietic tissue or circulating stem-cells/stem-like cells. Ambrosone et al. (224) investigated the effects of engineered nanoparticles (EPs) on regeneration success and proliferation of stem-cells of *Hydra vulgaris* using the bromodeoxyuridine immunodetection assay. Also, gene expression of the transcription factor Hymyc1, involved in stem cell proliferation/differentiation, was monitored. Furthermore, the impact of EPs as well as of 4-methylumbelliferon) on hematopoietic organ and circulating hematopoietic stem cells of silkworm (*Bombyx mori*) larvae was investigated (225–227). The rate of hematopoiesis in hematopoietic organ *in vitro*, apoptosis of circulating hemocytes, and internalization of EPs by prohemocytes (227) referring to it as “hematopoiesis toxicity” were reported (226). These kinds of studies benefit from the extensive knowledge of hemocyte proliferation and of the final steps of hemocyte maturation in the silkworm (228). Similarly, Oweson et al. (229) reported the effects of manganese on circulating immune cells of the common sea star *A. rubens*. Manganese induces the proliferation of the cells of the coelomic epithelium, a putative hematopoietic organ of echinoderms (see above), and increases the number of circulating coelomocytes. At the same time, coelomocytes showed stress response and their phagocytic capacity was negatively affected. Such findings can be considered as a building block in the understanding of the interplay between immune and stem cells in invertebrate species. The complex “defence” machinery used by them to face external stressors, including exposure to pollutants clearly identifies the involvement of both in the organism’s response and ability to recover and evolve. Taking into account the increasing environmental changes faced by aquatic invertebrate species in their natural environment and the variety of stressors to which they must adapt to survive, greater knowledge of the interplay between the immune system and stem cells would be useful for predicting new future scenarios and identify those species more prone to deal with it (**Figure 8**).

## Stem and Immune Cells Open Queries New Tools, and Future Perspectives

Research on aquatic invertebrate immunity and stem cells suffers from the scarce consideration of this group of animals as model organisms for biological research, with few notable exceptions represented by *Hydra* and planarians. Moreover, the lack of suitable *in vitro* models as well as of specific protocols for cell identification and cell culture limit their application also in ecotoxicity. Here below, we present some critical points that deserve consideration in the near future and can lead to rapid and significant advancements if tackled correctly.

### Identification of Stem and Immune Cells

**Figure 9:**
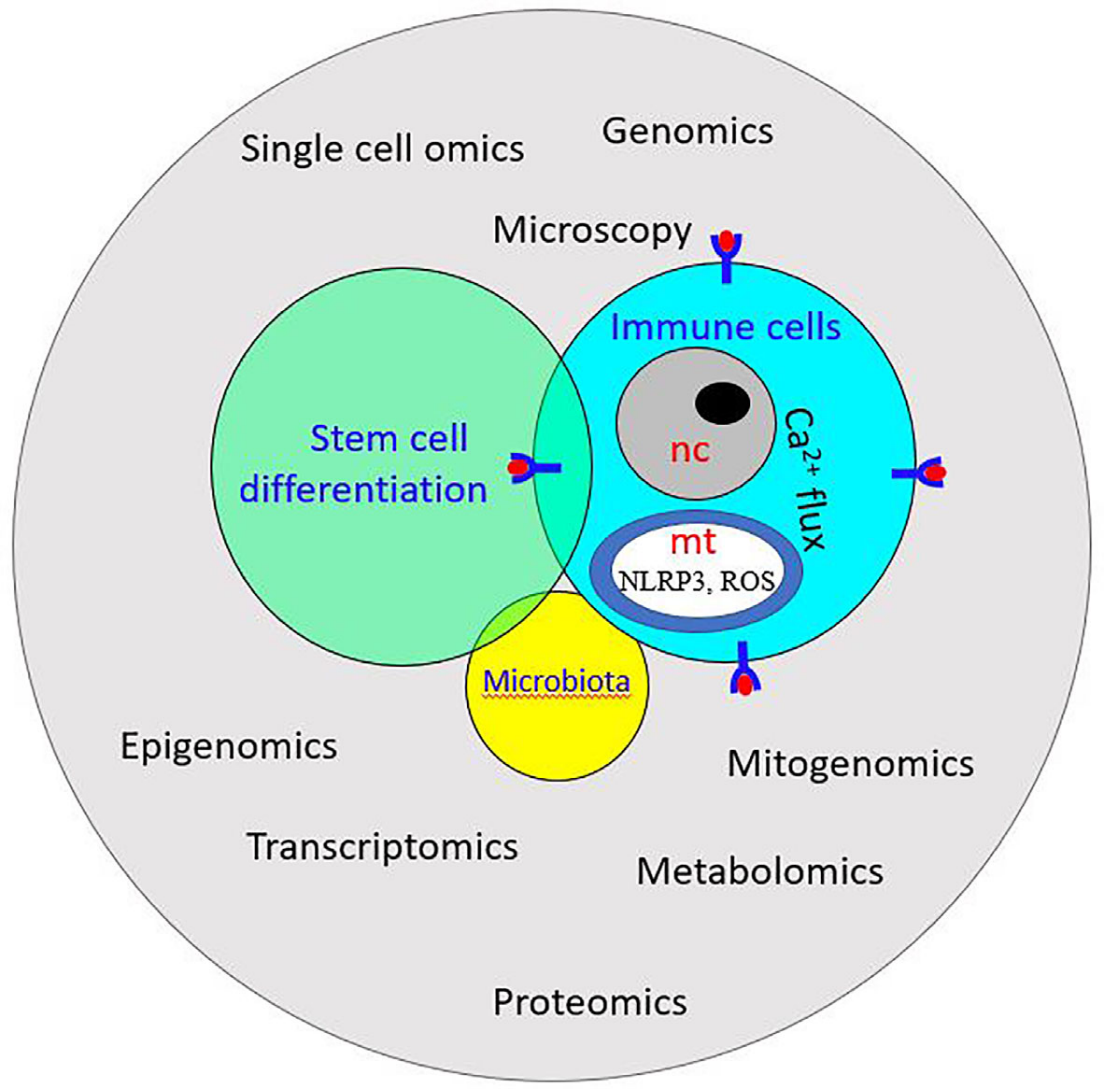
Schematic illustration of system biology: it exploits -omics technology to study the interplay among stem cells, immune cells, and microbiota. ROS, Reactive oxygen species; NLRP3, NOD-like receptor P3; nc, nucleus; mt, mitochondrion.

The paucity of available molecular markers to precisely identify stem and immune cells in aquatic invertebrates is still a limitation. Recently some progress has been made as for instance, in mussel hemocytes. Freshly isolated hemocytes from the mussel *M. galloprovincialis* have been shown to be highly responsive to mammalian stem cell growth factor in terms of both increased proliferation, and changes in immunoreactivity to the HSC marker CD117, or Kit-ligand receptor [(230); Canesi, personal communication] (**Figure 9**).

Up to now, light and electron microscopy have been the major methods for the identification of stem and immune cells of aquatic invertebrates. Light microscopy could be associated with immunohistochemistry or *in situ* hybridization to reveal the location of enzymatic activities or molecular markers. In addition, differential interference contrast and phase-contrast microscopy were also used for cell characterization (231–235). Scanning electron microscopy (SEM) was successfully applied to distinguish various hemocyte populations in aquatic invertebrates, as well as visualize the putative stem cells of some organisms (232, 236–239). Holland and Somorjai (240) used serial block-face scanning electron microscopy to distinguish the population of stem cells in invertebrate chordate.

Despite demanding sample preparation and time-consuming inspection of samples, transmission electron microscopy is the most widespread technique for the study of the ultrastructure of invertebrate circulating cell and putative stem cells (231, 232, 236, 241–250). However, new imaging technologies are emerging, such as optical coherence tomography, optical coherence phase microscope, microcirculation imaging, confocal reflectance microscopy, super-resolution microscopy, microcomputed tomography, FIB/SEM milling and subsurface imaging, radionuclide imaging that, if applied to aquatic invertebrates, can lead to a finer description of cell morphology and better identification and discrimination of stem and immune cells.

In crustaceans, the identification of different hemocyte lineages based on their morphology (251) was upgraded by studying the expression of some hematopoietic transcription factors (GATA, RUNX) and signal pathway molecules (JAK/STAT, Notch) (252). In addition, specific protein markers have been used for the identification of semigranulocytes (Kazal-type proteinase inhibitor and crustacean hematopoietic factor) and granulocytes (superoxide dismutase and mannan-binding lectin) (253, 254).

Homologues of the vertebrate stemness genes have been identified in many aquatic invertebrates studied so far (255–258) and were used as stem cell markers in various species (259–265). Furthermore, the application of flow cytometry, together with different markers of stem cells and hematopoietic cells, to circulating hemocytes/coelomocytes or to dissociated cells from early larval stages, can represent a powerful tool for investigating stem cells and innate immunity in adult and developing aquatic invertebrates. Recently, using flow cytometry sorting, whole-transcriptome sequencing, and diverse transplantation essays, Rosental et al. (266) identified hematopoietic stem cells and hematopoietic niches in the tunicate *B. schlosseri*. This study revealed that the subendostylar sinus is a hematopoietic stem cell niche while its molecular signature further suggests that the vertebrate hematopoietic bone marrow niche evolved from an organ resembling the *B. schlosseri* endostyle (266). The comparison of the HSC molecular signature during embryogenesis and blastogenesis, showed a similar early/late pattern of the HSC-associated gene enrichment, suggesting similar molecular dynamics of HSC development in embryogenesis and blastogenesis in *B. schlosseri* (137).

### *In Vitro* Cell Cultures

Although many efforts have been spent over many years of research, no established cell line from aquatic invertebrates is available today. This represents a serious obstacle to the full elucidation of the stem cell and immune cell properties in this group of animals and to promote their application in various disciplines (267, 268). The main limitations in cell harvesting lie in the *in vitro* requirements which leads to the failure of most invertebrate primary cultures within 24-72 h post cell isolation (7, 267), a topic that has been recently tested from an experimental point of view (269). However, some progress has been made as reported below. Conkling et al. (270) were able to keep in culture dissociated sponge cells for more than one month observing a significant increase in their number, Munroe et al. (271) have developed a genetic algorithm as an optimization tool for the development of sponge cell culture media, and efforts have been invested in studies on the establishment of sponge transgenesis (272). Ventura et al. (273) succeeded in maintaining primary cell cultures from dissociated tentacle cells of the cnidarian *Anemonia viridis* for one month. In crustaceans, long-term primary cultures of crayfish HPT cells were possible with the use of astakine, a new invertebrate cytokine directly involved in hematopoiesis, thus allowing the study of the molecular mechanism involved in the production of hemocytes (84, 252, 274). Transfection methods were also studied on the hematopoietic primary culture system from *Cherax quadricarinatus* (275) and efforts were invested in long-term ostracod epidermal cultures (276).

Recently, a method for the harvesting and short-/long-term maintenance of primary immune cells from sea urchin *Paracentrotus lividus* has been proposed (277). This method uses the coelomocyte culture medium, containing a high-affinity Ca^2+^ chelator, for short-term culture (≤48 h), and the artificial seawater as the master medium that maintains cell survival and *in vitro*-*ex vivo* physiological homeostasis over 2 weeks. Such method could unravel complex cellular phenomena and is pivotal in establishing the sea urchin invitrome (107, 108).

### ‘-Omics’-Based Technologies

Microscopy and molecular analysis of aquatic invertebrate stem-cell behavior can be fostered by the -omics tools pioneered for studying regeneration and immune responses. The last two decades have seen the generation of draft genomes of various aquatic invertebrate species (163, 278–293). The increasing number of data has been accompanied by increasing quality of assemblies and annotations, for example in oysters (294) and mussels (295). Studies on the genome of selected species are contributing to elucidate the molecular mechanisms underlying the processes of proliferation and differentiation of immune cells from stem cells. Moreover, the application of transcriptomic approaches to early developmental stages is helping to unravel the molecular signature of undifferentiated cells, including the expression of stem cell markers, transcription factors, and epigenetic modifications. For instance, Mao et al. (296) reported transcriptomic evidence of the molecular basis underlying functional differentiation of hemocytes in the bivalve *Crassostrea* sp., whereas Söderhäll and Junkunlo (297) recently reported a complete proteomic analysis from purified cell types from the crayfish HPT, the anterior proliferation center. This last work allowed the detection of several cell type-specific proteins and new putative biomarkers within the crayfish hematopoietic lineage that can be used to increase the understanding of how the differentiation process is regulated, are described.

Besides the nuclear genome, the mitochondrial genome’s omics approach can provide further information on the relationships between stem and immune cells. The release of DNA and/or reactive oxygen species by mitochondria represents a signal influencing immune cell transcription (298). Mitochondria can also regulate immunity through the alterations of metabolic pathways and are associated with NLRP3 inflammasome activation (299): mitochondria-induced transcriptional changes can lead to entirely different outcomes in immune cells (300). Mitochondria are also essential for stem cell plasticity (301) and it has been reported that mitochondria have a role in deciding stem cell fate by keeping the records of divisional histories (302, 303). Indeed, stem cell fate decisions are controlled by a complex balance between mitochondrial-nuclear interactions and Ca^2+^ flux that further modulates mitochondrial features during proliferation, metabolism, differentiation, and apoptosis (302, 304, 305).

### Single-Cell Transcriptomics

Stem and immune cells live in a complex environment aimed at protecting tissue integrity and homeostasis upon changes in functional demands; they have various genetic backgrounds and are endowed with disparate receptors on their plasma membrane (306). All these features are under the control of their genome, whose expression varies upon cell type. Today, it is possible to associate each cell with its “single-cell transcriptomics” (SCT) profile. Current SCT studies offer unprecedented opportunities to trace the evolutionary origins of every cell within a tissue/organ and reveal their activation pathways (258) and encompass a broad spectrum of organisms and cell types (307–316). SCT approaches can be used to elucidate how stem cells differentiate into the set of cells that make the body of the larval/adult organisms (258, 317, 318). The application of this technique to stem and immune cells of aquatic invertebrates can provide researchers with valuable information on transcriptome trajectories, regulatory differentiation cascades, and common gene networks underlying cell differentiation/activation during development and upon nonself recognition and response.

### Epigenetics Modifications

Cytosine methylation is one of the main covalent base modifications in eukaryotic genomes. It is involved in the epigenetic regulation of gene expression in a cell-type-specific manner, is reversible, and can remain stable through cell division. In mammals, epigenetic changes associated with stem cell self-renewal and differentiation (319, 320) as well as with the activation of immune cells (321) have been reported. Whereas invertebrates such as *Drosophila melanogaster* and *Caenorhabditis elegans* do not exhibit cytosine methylation and consequently do not have CpG rich and poor regions but rather a steady CpG frequency over the genome (322), some studies provided evidence of epigenetic modification in planarian stem cells (323) and in tissues of buds and regenerating zooids in colonial tunicates (324), but no investigation was addressed to aquatic invertebrate immune cells. Therefore, the study of histone methylation patterns can provide relevant information on its role in the maintenance of stem cell identity by chromatin remodeling and transcriptional control of pluripotency genes as well as in immune cell activation. In addition, the techniques of chromatin immunoprecipitation followed by sequencing (ChIP-Seq) for genome-wide profiling of DNA-binding proteins, histone modifications, or nucleosomes offer the possibility of a better understanding of the importance of chromatin modification in stem cell differentiation and immune cell activation. This can elucidate the gene regulatory networks characterizing the interplay between the transcriptome and the epigenome (325–327) in the cross-talks between stem and immune cells.

### Stem Cells, Immune Cells, and Gut Microbiota

It is widely accepted that the gut community of microorganisms has beneficial effects on animals and host-microbiome interactions contribute to the general organism homeostasis. Various data suggest the importance of gut microbiota in shaping an organism’s immune system/response (328–331). The opposite is also true: the immune system influences the microbial community in the gut (332). A clear demonstration of the close relationships among gut microorganisms and immune cells in humans comes from the observation that patients with inflammatory bowel disease are characterized by restricted biodiversity and unbalanced bacterial composition in their intestines associated with immune dysregulation (333, 334). Gut microbiota is also important for the establishment of the innate immune memory or immune priming in both vertebrates and invertebrates (331, 335, 336). Stem cell activity is also influenced by the gut microbiota, although few researchers focused on this (337, 338). Studies on the gut microbiota in aquatic invertebrates are still at the very beginning (339–343). However, since the gut microbiota of invertebrates is simpler than that of vertebrates (341), the former can be used as a suitable model to study its intimate dialogue with stem and immune cells. Indeed, Arnold et al. (56) reported evidence that innate immune responses and the machinery required for regeneration (which includes neoblasts) are modulated by the shift in gut microbial composition in the planarian *Schimdtea mediterranea*. Liberti et al. (344) recently provided an exhaustive review on the interaction between the immune system and the gut microbiota in the solitary ascidian *Ciona robusta*, but no data are available on the modulation of the stem cell activity by the microorganisms hosted in the gut.

### Systems Biology: Multi-Omics Approach

System biology is the integrative study of biological systems. It exploits the high volume of data from genomics, proteomics, metabolomics, and other omics- technologies and combines mathematical and computational models to characterize functional changes across different contexts (345, 346). Studies using a multi-omics approach integrating metagenomics, metatranscriptomics, metaproteomics and metabolomics data are already ongoing to clarify the molecular basis of some human diseases (347, 348). As far as aquatic invertebrates are concerned, multi–omics approaches, involving bioinformatics tools for the analysis of high throughput sequencing data, database designs for proteomics and metabolomics data, use of algorithms to detect transcriptional and translational changes is a new venture that will certainly help researchers in understanding their biological systems. A multi-omics approach was recently followed for studying the interaction between the host and the gut microbiota in the solitary ascidian *C. robusta* (344). Another aspect of system biology is the combination of omics-based data and morphological data. For instance, in the compound ascidian *B. schlosseri*, transcriptomic data were combined with multiple microscopy techniques to investigate the changes in molecular and morphological signatures of cells during embryogenesis and blastogenesis (314). This comprehensive approach will certainly help in identifying differentiation/activation pathways for stem and immune cells.

## Conclusion

Our review presents the *state-of-the-art* of the current knowledge on the relationship between stem cells and the immune system in selected aquatic invertebrate phyla. We point out the need to bridge two seemingly disparate disciplines, stem cell biology and the immune system research. The need to integrate current knowledge, methodological tools and concepts to provide a more holistic understanding of how organisms maintain and restore homeostasis using stem cells and immune system is underlined. Here we propose aquatic invertebrates as suitable models to deeply investigate the interplay between stem and immune cells, starting by addressing the basic equations in biology and further applying the acquired knowledge in other fields as ecotoxicology, biotechnology, and medicine. A parallel to vertebrates is presented to demonstrate how these two fields of research can be interwound.

The integrated analysis of existing information needs to be coupled with the generation of new data and the new tools available will allow cross-species extrapolations. The biological read-across can only be achieved by understanding similarities and differences in biological pathways through the involvement of a variety of species.

## Author Contributions

LB - substantial contributions to the conception, and drafting the work, revising it critically for important intellectual content. AK- substantial contributions to the conception and drafting the work, revising it critically for important intellectual content. AS - conception and drafting the work. LR - conception and drafting part of the work. LM - conception and drafting part of the work. BuR - conception and drafting, AR - conception and drafting. AV - provide approval for publication of the content. BeR - provide approval for publication of the content. LC - conception and drafting. CA - conception of the work. AP - conception of the work. BT - provide approval for publication of the content. AJ - provide approval for publication of the content. AD - provide approval for publication of the content. SN - provide approval for publication of the content. MS - conception and drafting part of the work. IC - provide approval for publication of the content. DD - substantial contributions to the conception, and drafting the work, revising it critically for important intellectual content. All authors contributed to the article and approved the submitted version.

## Funding

This study is supported by the European Cooperation in Science & Technology program (EU COST). COST Action 16203 MARISTEM: “Stem cells of marine/aquatic invertebrates: from basic research to innovative applications”. In addition, the authors would like to acknowledge also to EU H2020 project PANDORA (GA 671881). BR and AV (Irv Weissman’s laboratory) are supported by a grant from the United States-Israel Binational Science Foundation (BSF no. 2015012). The work of BR was supported by Israel Science Foundation (ISF) grant number 1416/19 and by HFSP Research Grant, RGY0085/2019.

## Conflict of Interest

The authors declare that the research was conducted in the absence of any commercial or financial relationships that could be construed as a potential conflict of interest.

## Abbreviations

ASC, Adult stem cell; DIC, Differential interference contrast; ENF, En1-lineage-naive fibroblast; ENP, En1-lineage-past fibroblast; EP, Engineered nanoparticle; HPT, Hematopoietic tissue; HSC, Hematopoietic stem cell; ISH, *In situ* hybridization; LPS, Lipopolysaccharide; MC, Morula cell; ROS, reactive oxygen species; SCT, Single cell transcriptomics; SEM, Scanning electron microscopy.

## References

[1] Weissman IL Shizuru JA. The Origins of the Identification and Isolation of Hematopoietic Stem Cells, and Their Capability to Induce Donor-Specific Transplantation Tolerance and Treat Autoimmune Diseases. Blood (2008) 112:3543–53. 10.1182/blood-2008-08-078220 PMC2574516 18948588

[2] Fagan MB. Philosophy of Stem Cell Biology - An Introduction. Philos Compass (2013) 8:1147–58. 10.1111/phc3.12088

[3] Morin E. On Complexity: Advances in Systems Theory, Complexity, and the Human Sciences. Cresskill, New Jersey: Hampton Press (2008).

[4] McLeod CJ Wang L Wong C Jones DL. Stem Cell Dynamics in Response to Nutrient Availability. Curr Biol (2010) 20:2100–5. 10.1016/j.cub.2010.10.038 PMC3005562 21055942

[5] Nakada D Levi BP Morrison SJ. Integrating Physiological Regulation With Stem Cell and Tissue Homeostasis. Neuron (2011) 70:703–18. 10.1016/j.neuron.2011.05.011 PMC4521627 21609826

[6] Odegaard JI Chawla A. The Immune System as a Sensor of the Metabolic State. Immunity (2013) 38:644–54. 10.1016/j.immuni.2013.04.001 PMC3663597 23601683

[7] Rosner A Armengaud J Ballarin L Barnay-Verdier S Cima F Coelho AV. Stem Cells of Aquatic Invertebrates as an Advanced Tool for Assessing Ecotoxicological Impacts. Sci Total Environ (2021) 771:144565. 10.1016/j.scitotenv.2020.144565 33736145

[8] Ballarin L Rinkevich B Bartscherer K Burzynski A Cambier S Cammarata M. Maristem—Stem Cells of Marine/Aquatic Invertebrates: From Basic Research to Innovative Applications. Sustainability (2018) 10:526. 10.3390/su10020526

[9] Fagan MB. The Search for the Hematopoietic Stem Cell: Social Interaction and Epistemic Success in Immunology. Stud Hist Philos Sci Part C Stud Hist Philos Biol BioMed Sci (2007) 38:217–37. 10.1016/j.shpsc.2006.12.010 17324815

[10] Welniak LA Blazar BR Murphy WJ. Immunobiology of Allogeneic Hematopoietic Stem Cell Transplantation. Annu Rev Immunol (2007) 25:139–70. 10.1146/annurev.immunol.25.022106.141606 17129175

[11] Boraschi D Alijagic A Auguste M Barbero F Ferrari E Hernadi S. Addressing Nanomaterial Immunosafety by Evaluating Innate Immunity Across Living Species. Small (2020) 162000598. 10.1002/smll.202000598 32363795

[12] Johansson MW Keyser P Sritunyalucksana K Söderhäll K. Crustacean Haemocytes and Haematopoiesis. Aquaculture (2000) 191:45–52. 10.1016/S0044-8486(00)00418-X

[13] Van De Braak CBT Botterblom MHA Liu W Taverne N van der Knaap WPW Rombout JHWM. The Role of the Haematopoietic Tissue in Haemocyte Production and Maturation in the Black Tiger Shrimp (*Penaeus monodon*). Fish Shellfish Immunol (2002) 12:253–72. 10.1006/fsim.2001.0369 11931020

[14] Noonin C Lin X Jiravanichpaisal P Söderhäll K Söderhäll I. Invertebrate Hematopoiesis: An Anterior Proliferation Center as a Link Between the Hematopoietic Tissue and the Brain. Stem Cells Dev (2012) 21:3173–86. 10.1089/scd.2012.0077 22564088

[15] Hartenstein V. Blood Cells and Blood Cell Development in the Animal Kingdom. Annu Rev Cell Dev Biol (2006) 22:677–712. 10.1146/annurev.cellbio.22.010605.093317 16824014

[16] Pila EA Sullivan JT Wu XZ Fang J Rudko SP Gordy MA. Haematopoiesis in Molluscs: A Review of Haemocyte Development and Function in Gastropods, Cephalopods and Bivalves. Dev Comp Immunol (2016) 58:119–28. 10.1016/j.dci.2015.11.010 PMC4775334 26592965

[17] Grimaldi A. Origin and Fate of Hematopoietic Stem Precursor Cells in the Leech Hirudo medicinalis. Invertebr Surviv J (2016) 13:257–68. 10.25431/1824-307x/isj.v13i1.257-268

[18] Voskoboynik A Soen Y Rinkevich Y Rosner A Ueno H Reshef R. Identification of the Endostyle as a Stem Cell Niche in a Colonial Chordate. Cell Stem Cell (2008) 3:456–64. 10.1016/j.stem.2008.07.023 PMC4126901 18940736

[19] Golconda P Buckley KM Reynolds CR Romanello JP Smith LC. The Axial Organ and the Pharynx Are Sites of Hematopoiesis in the Sea Urchin. Front Immunol (2019) 10:870. 10.3389/fimmu.2019.00870 31105697 PMC6494969

[20] Abnave P Ghigo E. Role of the Immune System in Regeneration and Its Dynamic Interplay With Adult Stem Cells. Semin Cell Dev Biol (2019) 87:160–8. 10.1016/j.semcdb.2018.04.002 29635020

[21] Lay K Yuan S Gur-Cohen S Miao Y Han T Naik S. Stem Cells Repurpose Proliferation to Contain a Breach in Their Niche Barrier. Elife (2018) 7:e41661. 10.7554/eLife.41661 30520726 PMC6324878

[22] Castillo M Liu K Bonilla L Rameshwar P. The Immune Properties of Mesenchymal Stem Cells. Int J BioMed Sci (2007) 3:76–80. 23675026 PMC3614633

[23] Dela Rosa O Dalemans W Lombardo E. Toll-Like Receptors as Modulators of Mesenchymal Stem Cells. Front Immunol (2012) 3:182. 10.3389/fimmu.2012.00182 22783256 PMC3387651

[24] De Laval B Maurizio J Kandalla PK Brisou G Simonnet L Huber C. C/Ebpβ-Dependent Epigenetic Memory Induces Trained Immunity in Hematopoietic Stem Cells. Cell Stem Cell (2020) 26:657–74. 10.1016/j.stem.2020.01.017. e8. 32169166

[25] Naik S Larsen SB Cowley CJ Fuchs E. Two to Tango: Dialog Between Immunity and Stem Cells in Health and Disease. Cell (2018) 175:908–20. 10.1016/j.cell.2018.08.071 PMC6294328 30388451

[26] Mescher AL Neff AW. Regenerative Capacity and the Developing Immune System. Adv Biochem Eng Biotechnol (2005) 93:39–66. 10.1007/b99966 15791943

[27] Julier Z Park AJ Briquez PS Martino MM. Promoting Tissue Regeneration by Modulating the Immune System. Acta Biomater (2017) 53:13–28. 10.1016/j.actbio.2017.01.056 28119112

[28] Godwin JW Pinto AR Rosenthal NA. Chasing the Recipe for a Pro-Regenerative Immune System. Semin Cell Dev Biol (2017) 61:71–9. 10.1016/j.semcdb.2016.08.008 PMC5338634 27521522

[29] Alibardi L. Tail Regeneration in Lepidosauria as an Exception to the Generalized Lack of Organ Regeneration in Amniotes. J Exp Zool Part B Mol Dev Evol (2019) 336:145–64. 10.1002/jez.b.22901 31532061

[30] Godwin JW Pinto AR Rosenthal NA. Macrophages Are Required for Adult Salamander Limb Regeneration. Proc Natl Acad Sci (2013) 110:9415–20. 10.1073/pnas.1300290110 PMC3677454 23690624

[31] Kanao T Miyachi Y. Lymphangiogenesis Promotes Lens Destruction and Subsequent Lens Regeneration in the Newt Eyeball, and Both Processes can be Accelerated by Transplantation of Dendritic Cells. Dev Biol (2006) 290:118–24. 10.1016/j.ydbio.2005.11.017 16343476

[32] Petrie TA Strand NS Yang C-T Rabinowitz JS Moon RT. Macrophages Modulate Adult Zebrafish Tail Fin Regeneration. Development (2015) 141:2581–91. 10.1242/dev.120642 PMC4067955 24961798

[33] Kokaia Z Martino G Schwartz M Lindvall O. Cross-Talk Between Neural Stem Cells and Immune Cells: The Key to Better Brain Repair? Nat Neurosci (2012) 15:1078–87. 10.1038/nn.3163 22837038

[34] Carpentier PA Palmer TD. Immune Influence on Adult Neural Stem Cell Regulation and Function. Neuron (2009) 64:79–92. 10.1016/j.neuron.2009.08.038 19840551 PMC2789107

[35] Ekdahl CT Claasen JH Bonde S Kokaia Z Lindvall O. Inflammation Is Detrimental for Neurogenesis in Adult Brain. Proc Natl Acad Sci USA (2003) 100:13632–7. 10.1073/pnas.2234031100 PMC263865 14581618

[36] Monje ML Toda H Palmer TD. Inflammatory Blockade Restores Adult Hippocampal Neurogenesis. Science (2003) 302:1760–5. 10.1126/science.1088417 14615545

[37] Beltz BS Zhang Y Benton JL Sandeman DC. Adult Neurogenesis in the Decapod Crustacean Brain: A Hematopoietic Connection? Eur J Neurosci (2011) 34:870–83. 10.1111/j.1460-9568.2011.07802.x PMC3178839 21929622

[38] Yin H Price F Rudnicki MA. Satellite Cells and the Muscle Stem Cell Niche. Physiol Rev (2013) 93:23–67. 10.1152/physrev.00043.2011 23303905 PMC4073943

[39] Rinkevich B. Primitive Immune Systems: Are Your Ways My Ways? Immunol Rev (2004) 198:25–35. 10.1111/j.0105-2896.2004.0114.x 15199952

[40] Gamulin V Rinkevich B Schäcke H Kruse M Müller IM Müller WEG. Cell Adhesion Receptors and Nuclear Receptors Are Highly Conserved From the Lowest Metazoa (Marine Sponges) to Vertebrates. Biol Chem Hoppe Seyler (1994) 375:583–8. 10.1515/bchm3.1994.375.9.583 7840899

[41] Schäcke H Schröder HC Gamulin V Rinkevich B Mülle IM Müller WEG. Molecular Cloning of a Tyrosine Kinase Gene From the Marine Sponge Geodia Cydonium: A New Member Belonging to the Receptor Tyrosine Kinase Class II Family. Mol Membr Biol (1994) 11:101–7. 10.3109/09687689409162227 7920862

[42] Oren M Douek J Fishelson Z Rinkevich B. Identification of Immune-Relevant Genes in Histoincompatible Rejecting Colonies of the Tunicate. Botryllus schlosseri Dev Comp Immunol (2007) 31:889–902. 10.1016/j.dci.2006.12.009 17287019

[43] Oren M Escande ML Paz G Fishelson Z Rinkevich B. Urochordate Histoincompatible Interactions Activate Vertebrate-Like Coagulation System Components. PloS One (2008) 3:e3123. 10.1371/journal.pone.0003123 18769590 PMC2527998

[44] Oren M Paz G Douek J Rosner A Amar KO Rinkevich B. Marine Invertebrates Cross Phyla Comparisons Reveal Highly Conserved Immune Machinery. Immunobiology (2013) 218:484–95. 10.1016/j.imbio.2012.06.004 22884351

[45] Mueller WA Rinkevich B. Cell Communication-mediated Nonself-Recognition and -Intolerance in Representative Species of the Animal Kingdom. J Mol Evol (2020) 88:482–500. 10.1007/s00239-020-09955-z 32572694

[46] Parisi MG Parrinello D Stabili L Cammarata M. Cnidarian Immunity and the Repertoire of Defense Mechanisms in Anthozoans. Biology (2020) 9:283. 10.3390/biology9090283 PMC7563517 32932829

[47] Magor BG De Tomaso A Rinkevich B Weissman IL. Allorecognition in Colonial Tunicates: Protection Against Predatory Cell Lineages? Immunol Rev (1999) 167:69–79. 10.1111/j.1600-065X.1999.tb01383.x 10319252

[48] Rinkevich B. Invertebrates Versus Vertebrates Innate Immunity: In the Light of Evolution. Scand J Immunol (1999) 50:456–60. 10.1046/j.1365-3083.1999.00626.x 10564546

[49] Peiris TH Hoyer KK Oviedo NJ. Innate Immune System and Tissue Regeneration in Planarians: An Area Ripe for Exploration. Semin Immunol (2014) 26:295–302. 10.1016/j.smim.2014.06.005 25082737 PMC4171206

[50] Alessandra S Rossi L. Planarian Stem Cell Heterogeneity. Adv Exp Med Biol (2019) 1123:39–54. 10.1007/978-3-030-11096-3_4 31016594

[51] Reddien PW. The Cellular and Molecular Basis for Planarian Regeneration. Cell (2018) 175:327–45. 10.1016/j.cell.2018.09.021 PMC7706840 30290140

[52] Rossi L Salvetti A. Planarian Stem Cell Niche, the Challenge for Understanding Tissue Regeneration. Semin Cell Dev Biol (2019) 87:30–6. 10.1016/j.semcdb.2018.03.005 29534938

[53] Morita M. Structure and Function of the Reticular Cell in the Planarian Dugesia dorotocephala. Hydrobiol Int J Aquat Sci (1995) 305:189–96. 10.1007/BF00036385

[54] Scimone ML Wurtzel O Malecek K Fincher CT Oderberg IM Kravarik KM. foxF-1 Controls Specification of Non-Body Wall Muscle and Phagocytic Cells in Planarians. Curr Biol (2018) 28:3787–801. 10.1016/j.cub.2018.10.030. e6. PMC6411049 30471994

[55] Serhan CN Dalli J Karamnov S Choi A Park C-K Xu Z-Z. Macrophage Proresolving Mediator Maresin 1 Stimulates Tissue Regeneration and Controls Pain. FASEB J (2012) 26:1755–65. 10.1096/fj.11-201442 PMC3316905 22253477

[56] Arnold CP Shane Merryman M Harris-Arnold A McKinney SA Seidel CW Loethen S. Pathogenic Shifts in Endogenous Microbiota Impede Tissue Regeneration *Via* Distinct Activation of TAK1/MKK/p38. Elife (2016) 5:e16793. 10.7554/eLife.16793 27441386 PMC4993586

[57] Altincicek B Vilcinskas A. Comparative Analysis of Septic Injury-Inducible Genes in Phylogenetically Distant Model Organisms of Regeneration and Stem Cell Research, the Planarian *Schmidtea mediterranea* and the Cnidarian *Hydra vulgaris* . Front Zool (2008) 5:1–12. 10.1186/1742-9994-5-6 18439314 PMC2386466

[58] Abnave P Mottola G Gimenez G Boucherit N Trouplin V Torre C. Screening in Planarians Identifies MORN2 as a Key Component in LC3-associated Phagocytosis and Resistance to Bacterial Infection. Cell Host Microbe (2014) 16:338–50. 10.1016/j.chom.2014.08.002 25211076

[59] Kangale LJ Raoult D Fournier PE Abnave P Ghigo E. Planarians (Platyhelminthes)—an Emerging Model Organism for Investigating Innate Immune Mechanisms. Front Cell Infect Microbiol (2021) 11:619081. 10.3389/fcimb.2021.619081 33732660 PMC7958881

[60] Wang IE Wagner DE Reddien PW. Clonal Analysis of Planarian Stem Cells by Subtotal Irradiation and Single-Cell Transplantation. Methods Mol Biol (2018) 1774:479–95. 10.1007/978-1-4939-7802-1_20 29916173

[61] Hayashi T Agata K. A Subtractive Facs Method for Isolation of Planarian Stem Cells and Neural Cells. Methods Mol Biol (2018) 1774:467–78. 10.1007/978-1-4939-7802-1_19 29916172

[62] Gosling E. Marine Bivalve Molluscs. Chichestwer, UK: John Wiley & Sons (2015). 10.1002/9781119045212

[63] Gerdol M Gomez-Chiarri M Castillo MG Figueras A Fiorito G Moreira R. Immunity in Molluscs: Recognition and Effector Mechanisms, With a Focus on Bivalvia. In: Cooper E, editor. Advances in Comparative Immunology. Cham: Springer (2018). p. 225–341. 10.1007/978-3-319-76768-0_11

[64] García-García E Prado-Álvarez M Novoa B Figueras A Rosales C. Immune Responses of Mussel Hemocyte Subpopulations Are Differentially Regulated by Enzymes of the PI 3-K, PKC, and ERK Kinase Families. Dev Comp Immunol (2008) 32:637–53. 10.1016/j.dci.2007.10.004 18045688

[65] Jemaa M Morin N Cavelier P Cau J Strub JM Delsert C. Adult Somatic Progenitor Cells and Hematopoiesis in Oysters. J Exp Biol (2014) 217:3067–77. 10.1242/jeb.106575 24948634

[66] Renwrantz L Siegmund E Woldmann M. Variations in Hemocyte Counts in the Mussel, *Mytilus edulis*: Similar Reaction Patterns Occur in Disappearance and Return of Molluscan Hemocytes and Vertebrate Leukocytes. Comp Biochem Physiol A Mol Integr Physiol (2013) 164:629–37. 10.1016/j.cbpa.2013.01.021 23384685

[67] Blier PU Abele D Munro D Degletagne C Rodriguez E Hagen T. What Modulates Animal Longevity? Fast and Slow Aging in Bivalves as a Model for the Study of Lifespan. Semin Cell Dev Biol (2017) 70:130–40. 10.1016/j.semcdb.2017.07.046 28778411

[68] Matozzo V Marin MG Cima F Ballarin L. First Evidence of Cell Division in Circulating Haemocytes From the Manila Clam *Tapes philippinarum* . Cell Biol Int (2008) 32:865–8. 10.1016/j.cellbi.2008.03.008 18440833

[69] Xia X Guan C Chen J Qiu M Qi J Wei M. Molecular Characterization of AwSox2 From Bivalve *Anodonta Woodiana*: Elucidating Its Player in the Immune Response. Innate Immun (2020) 26:381–97. 10.1177/1753425919897823 PMC7903536 31889462

[70] Li Y Song X Wang W Wang L Yi Q Jiang S. The Hematopoiesis in Gill and Its Role in the Immune Response of Pacific Oyster *Crassostrea gigas* Against Secondary Challenge With Vibrio Splendidus. Dev Comp Immunol (2017) 71:59–69. 10.1016/j.dci.2017.01.024 28159592

[71] Cima F Matozzo V. Proliferation and Differentiation of Circulating Haemocytes of *Ruditapes philippinarum* as a Response to Bacterial Challenge. Fish Shellfish Immunol (2018) 81:73–82. 10.1016/j.fsi.2018.07.010 29981883

[72] Auguste M Balbi T Ciacci C Canonico B Papa S Borello A. Shift in Immune Parameters After Repeated Exposure to Nanoplastics in the Marine Bivalve *Mytilus* . Front Immunol (2020) 11:426. 10.3389/fimmu.2020.00426 32351496 PMC7174705

[73] Dyachuk VA. Hematopoiesis in Bivalvia Larvae: Cellular Origin, Differentiation of Hemocytes, and Neoplasia. Dev Comp Immunol (2016) 65:253–7. 10.1016/j.dci.2016.07.019 27486682

[74] Giribet G Edgecombe GD. Reevaluating the Arthropod Tree of Life. Annu Rev Entomol (2012) 57:167–86. 10.1146/annurev-ento-120710-100659 21910637

[75] Hauton C. The Scope of the Crustacean Immune System for Disease Control. J Invertebr Pathol (2012) 110:251–60. 10.1016/j.jip.2012.03.005 22441033

[76] Vogt G. Hidden Treasures in Stem Cells of Indeterminately Growing Bilaterian Invertebrates. Stem Cell Rev Rep (2012) 8:305–17. 10.1007/s12015-011-9303-1 21785941

[77] Halcrow K Smith JC. Wound Closure in the Crab *Carcinus maenas* (L.). Can J Zool (1986) 64:2770–8. 10.1139/z86-401

[78] Uhrík B Rýdlová K Zacharová D. The Roles of Haemocytes During Degeneration and Regeneration of Crayfish Muscle Fibres. Cell Tissue Res (1989) 255:443–9. 10.1007/BF00224130 2924344

[79] Da Silva PGC De Abreu IS Cavalcante LA De Barros CM Allodi S. Role of Hemocytes in Invertebrate Adult Neurogenesis and Brain Repair. Invertebr Surviv J (2015) 12:142–54.

[80] Lin X Söderhäll I. Crustacean Hematopoiesis and the Astakine Cytokines. Blood (2011) 117:6417–24. 10.1182/blood-2010-11-320614 21444913

[81] Söderhäll K Cerenius L. Crustacean Immunity. Annu Rev Fish Dis (1992) 2:3–23. 10.1016/0959-8030(92)90053-Z

[82] Chaga O Lignell M Söderhäll K. The Haemopoietic Cells of the Freshwater Crayfish *Pacifastacus leniusculus* . Anim Biol (1995) 4:59–70.

[83] Martin GG Hose JE Choi M Provost R Omori G McKrell N. Organization of Hematopoietic Tissue in the Intermolt Lobster, *Homarus americanus* . J Morphol (1993) 216:65–78. 10.1002/jmor.1052160108 29865454

[84] Söderhäll I Bangyeekhun E Mayo S Söderhäll K. Hemocyte Production and Maturation in an Invertebrate Animal; Proliferation and Gene Expression in Hematopoietic Stem Cells of *Pacifastacus leniusculus* . Dev Comp Immunol (2003) 27:661–72. 10.1016/S0145-305X(03)00039-9 12798363

[85] Song CK Johnstone LM Schmidt M Derby CD Edwards DH. Social Domination Increases Neuronal Survival in the Brain of Juvenile Crayfish *Procambarus clarkii* . J Exp Biol (2007) 210:1311–24. 10.1242/jeb.02758 17401115

[86] Martynova M. Satellite Cells in the Crayfish Heart Muscle Functions as Stem Cells and Are Characterized by Molt-Dependent Behaviour. Zool Anz (1993) 230:181–90.

[87] Vogt G. Life-Cycle and Functional Cytology of the Hepatopancreatic Cells of *Astacus astacus* (Crustacea, Decapoda). Zoomorphology (1994) 114:83–101. 10.1007/BF00396642

[88] Benton JL Kery R Li J Noonin C Söderhäll I Beltz BS. Cells From the Immune System Generate Adult-Born Neurons in Crayfish. Dev Cell (2014) 30:322–33. 10.1016/j.devcel.2014.06.016 25117683

[89] Vogt G. Suitability of the Clonal Marbled Crayfish for Biogerontological Research: A Review and Perspective, With Remarks on Some Further Crustaceans. Biogerontology (2010) 11:643–69. 10.1007/s10522-010-9291-6 20582627

[90] Aurora AB Olson EN. Immune Modulation of Stem Cells and Regeneration. Cell Stem Cell (2014) 15:14–25. 10.1016/j.stem.2014.06.009 24996166 PMC4131296

[91] Díaz AC Sousa LG Petriella AM. Functional Cytology of the Hepatopancreas of *Palaemonetes Argentinus* (Crustacea, Decapoda, Caridea) Under Osmotic Stress. Braz Arch Biol Technol (2010) 53:599–608. 10.1590/S1516-89132010000300013

[92] Bottjer DJ Davidson EH Peterson KJ Cameron RA. Paleogenomics of Echinoderms. Science (2006) 314:956–60. 10.1126/science.1132310 17095693

[93] Smith LC Arizza V Barela Hudgell MA Barone G Bodnar AG Buckley KM. Echinodermata: The Complex Immune System in Echinoderms. In: Cooper E, editor. Advances in Comparative Immunology. Cham: Springer (2018). p. 409–501. 10.1007/978-3-319-76768-0_13

[94] Hamdoun A Epel D. Embryo Stability and Vulnerability in an Always Changing World. Proc Natl Acad Sci (2007) 104:1745–50. 10.1073/pnas.0610108104 PMC1794293 17264211

[95] Chia FS Xing J. Echinoderm Coelomocytes. Zool Stud (1996) 35:231–54.

[96] Candia Carnevali MD Bonasoro F. Microscopic Overview of Crinoid Regeneration. Microsc Res Tech (2001) 55:403–26. 10.1002/jemt.1187 11782071

[97] Pinsino A Matranga V. Sea Urchin Immune Cells as Sentinels of Environmental Stress. Dev Comp Immunol (2015) 49:198–205. 10.1016/j.dci.2014.11.013 25463510

[98] Andrade C Oliveira B Guatelli S Martinez P Simões B Bispo C. Characterization of Coelomic Fluid Cell Types in the Starfish *Marthasterias glacialis* Using a Flow Cytometry/Imaging Combined Approach. Front Immunol (2021) 12:641664. 10.3389/fimmu.2021.641664 33815394 PMC8013778

[99] Parrinello N. Cytotoxic Activity of Tunicate Hemocytes. Prog Mol Subcell Biol (1996) 15:190–217. 10.1007/978-3-642-79735-4_9 8963462

[100] Xing K Yang H Chen M. Morphological and Ultrastructural Characterization of the Coelomocytes in. Apostichopus japonicus Aquat Biol (2008) 2:85–92. 10.3354/ab00038

[101] Gorshkov AN Blinova MI Pinaev GP. Ultrastructure of Coelomic Epithelium and Coelomocytes of the Starfish *Asterias rubens* L. in Norm and After Wounding. Cell Tissue Biol (2009) 3:477–90. 10.1134/S1990519X09050113 19799349

[102] Smith LC Ghosh J Buckley KM Clow LA Dheilly NM Haug T. Echinoderm Immunity. Adv Exp Med Biol (2010) 708:260–301. 10.1007/978-1-4419-8059-5_14 21528703

[103] Franco CF Santos R Coelho AV. Proteome Characterization of Sea Star Coelomocytes - The Innate Immune Effector Cells of Echinoderms. Proteomics (2011) 11:3587–92. 10.1002/pmic.201000745 21751360

[104] Taguchi M Tsutsui S Nakamura O. Differential Count and Time-Course Analysis of the Cellular Composition of Coelomocyte Aggregate of the Japanese Sea Cucumber *Apostichopus japonicus* . Fish Shellfish Immunol (2016) 58:203–9. 10.1016/j.fsi.2016.06.060 27633669

[105] Sharlaimova N Shabelnikov S Bobkov D Martynova M Bystrova O Petukhova O. Coelomocyte Replenishment in Adult *Asterias Rubens*: The Possible Ways. Cell Tissue Res (2021) 383:1043–60. 10.1007/s00441-020-03337-z 33237478

[106] Alijagic A Benada O Kofroňová O Cigna D Pinsino A. Sea Urchin Extracellular Proteins Design a Complex Protein Corona on Titanium Dioxide Nanoparticle Surface Influencing Immune Cell Behavior. Front Immunol (2019) 10:2261. 10.3389/fimmu.2019.02261 31616433 PMC6763604

[107] Alijagic A Barbero F Gaglio D Napodano E Benada O Kofroňová O. Gold Nanoparticles Coated With Polyvinylpyrrolidone and Sea Urchin Extracellular Molecules Induce Transient Immune Activation. J Hazard Mater (2021) 402:123793. 10.1016/j.jhazmat.2020.123793 33254802

[108] Alijagic A Gaglio D Napodano E Russo R Costa C Benada O. Titanium Dioxide Nanoparticles Temporarily Influence the Sea Urchin Immunological State Suppressing Inflammatory-Relate Gene Transcription and Boosting Antioxidant Metabolic Activity. J Hazard Mater (2020) 384:121389. 10.1016/j.jhazmat.2019.121389 31639584

[109] Bossche JPV Jangoux M. Epithelial Origin of Starfish Coelomocytes. Nature (1976) 261:227–8. 10.1038/261227a0 1272394

[110] Holm K Dupont S Sköld H Stenius A Thorndyke M Hernroth B. Induced Cell Proliferation in Putative Haematopoietic Tissues of the Sea Star *Asterias rubens* (L). J Exp Biol (2008) 211:2551–8. 10.1242/jeb.018507 18689408

[111] Hernroth B Farahani F Brunborg G Dupont S Dejmek A Sköld HN. Possibility of Mixed Progenitor Cells in Sea Star Arm Regeneration. J Exp Zool Part B Mol Dev Evol (2010) 314 B:457–68. 10.1002/jez.b.21352 20700890

[112] Ferguson JC. Cell Production in the Tiedemann Bodies and Haemal Organs of the Starfish *Asterias forbesi* . Trans Am Microsc Soc (1966) 85:200. 10.2307/3224630

[113] Bachmann S Goldschmid A. The Echinoid Axial Complex and Tiedemann Bodies - the Different Pathways and Accumulation Sites of Coelomocytes With Regard to Waste Disposal in the Organism. In: Jangoux M, editor. Echinoderms: Present & Past. Proceeding of the European Colloquium on Echinoderms. Balkema: Rotterdam (1981).

[114] Sharlaimova N Shabelnikov S Petukhova O. Small Coelomic Epithelial Cells of the Starfish *Asterias rubens* L. That Are Able to Proliferate *In Vivo* and *In Vitro* . Cell Tissue Res (2014) 356:83–95. 10.1007/s00441-013-1766-8 24408073

[115] Moss G. Patterns of Bromodeoxyuridine Incorporation and Neuropeptide Immunoreactivity During Arm Regeneration in the Starfish *Asterias rubens* . Philos Trans R Soc B Biol Sci (1998) 353:421–36. 10.1098/rstb.1998.0220

[116] Candia Carnevali MD. Regeneration in Echinoderms: Repair, Regrowth and Cloning. ISJ-Invertebr Surviv J (2006) 3:64–76.

[117] Ben Khadra Y Sugni M Ferrario C Bonasoro F Oliveri P Martinez P. Regeneration in Stellate Echinoderms: Crinoidea, Asteroidea and Ophiuroidea. In: Kloc M Kubiak J, editors. Results and Problems in Cell Differentiation. Cham: Springer (2018). p. 285–320. 10.1007/978-3-319-92486-1_14 30083925

[118] García-Arrarás JE Valentín-Tirado G Flores JE Rosa RJ Rivera-Cruz A San Miguel-Ruiz JE. Cell Dedifferentiation and Epithelial to Mesenchymal Transitions During Intestinal Regeneration in *H. glaberrima* . BMC Dev Biol (2011) 11:1–18. 10.1186/1471-213X-11-61 22004330 PMC3207902

[119] Ferrario C Sugni M Somorjai IML Ballarin L. Beyond Adult Stem Cells: Dedifferentiation as a Unifying Mechanism Underlying Regeneration in Invertebrate Deuterostomes. Front Cell Dev Biol (2020) 8:587320. 10.3389/fcell.2020.587320 33195242 PMC7606891

[120] Piovani L Czarkwiani A Ferrario C Sugni M Oliveri P. Ultrastructural and Molecular Analysis of the Origin and Differentiation of Cells Mediating Brittle Star Skeletal Regeneration. BMC Biol (2021) 19:1–19. 10.1186/s12915-020-00937-7 33461552 PMC7814545

[121] Pinsino A Thorndyke MC Matranga V. Coelomocytes and Post-Traumatic Response in the Common Sea Star *Asterias rubens* . Cell Stress Chaperones (2007) 12:331–41. 10.1379/CSC-288.1 PMC2134795 18229452

[122] Delsuc F Tsagkogeorga G Lartillot N Philippe H. Additional Molecular Support for the New Chordate Phylogeny. Genesis (2008) 46:592–604. 10.1002/dvg.20450 19003928

[123] Hirose E. Ascidian Tunic Cells: Morphology and Functional Diversity of Free Cells Outside the Epidermis. Wiley Online Libr (2009) 128:83–96. 10.1111/j.1744-7410.2008.00153.x

[124] Zaniolo G. Histology of the Ascidian *Botryllus schlosseri* Tunic: in Particular, the Test Cells. Ital J Zool (1981) 48:169–78. 10.1080/11250008109439330

[125] Burighel P Cloney R. Urochordata: Ascidiacea. In: Harrison FW Ruppert EE, editors. Microscopic Anatomy of Invertebrates - Hemichordata, Chaetognatha and the Invertebrate Chordates. New York: Wiley-Liss, Inc (1997). p. 221–347.

[126] Di Bella MA Carbone MC De Leo G. Aspects of Cell Production in Mantle Tissue of *Ciona intestinalis* L. (Tunicata, Ascidiacea). Micron (2005) 36:477–81. 10.1016/j.micron.2005.01.007 15935306

[127] Cima F Franchi N Ballarin L. Origin and Functions of Tunicate Hemocytes. In: Malagoli D, editor. The Evolution of the Immune System: Conservation and Diversification. London: Elsevier (2016). p. 29–49. 10.1016/B978-0-12-801975-7.00002-5

[128] Franchi N Ballarin L. Immunity in Protochordates: The Tunicate Perspective. Front Immunol (2017) 8:674. 10.3389/fimmu.2017.00674 28649250 PMC5465252

[129] Manni L Zaniolo G Cima F Burighel P Ballarin L. *Botryllus schlosseri*: A Model Ascidian for the Study of Asexual Reproduction. Dev Dyn (2007) 236:335–52. 10.1002/dvdy.21037 17191252

[130] Manni L Anselmi C Cima F Gasparini F Voskoboynik A Martini M. Sixty Years of Experimental Studies on the Blastogenesis of the Colonial Tunicate *Botryllus schlosseri* . Dev Biol (2019) 448:293–308. 10.1016/j.ydbio.2018.09.009 30217596

[131] Blanchoud S Rutherford K Zondag L Gemmell NJ Wilson MJ. De Novo Draft Assembly of the *Botrylloides leachii* Genome Provides Further Insight Into Tunicate Evolution. Sci Rep (2018) 8:1–18. 10.1038/s41598-018-23749-w 29615780 PMC5882950

[132] Lauzon RJ Rinkevich B Patton CW Weissman IL. A Morphological Study of Nonrandom Senescence in a Colonial Urochordate. Biol Bull (2000) 198:367–78. 10.2307/1542692 10897450

[133] Lauzon RJ Ishizuka KJ Weissman IL. A Cyclical, Developmentally-Regulated Death Phenomenon in a Colonial Urochordate. Dev Dyn (1992) 194:71–83. 10.1002/aja.1001940109 1421521

[134] Lauzon RJ Patton CW Weissman IL. A Morphological and Immunohistochemical Study of Programmed Cell Death in *Botryllus schlosseri* (Tunicata, Ascidiacea). Cell Tissue Res (1993) 272:115–27. 10.1007/BF00323577 8386984

[135] Cima F Basso G Ballarin L. Apoptosis and Phosphatidylserine-Mediated Recognition During the Take-Over Phase of the Colonial Life-Cycle in the Ascidian *Botryllus schlosseri* . Cell Tissue Res (2003) 312:369–76. 10.1007/s00441-003-0738-9 12764610

[136] Ballarin L Menin A Tallandini L Matozzo V Burighel P Basso G. Haemocytes and Blastogenetic Cycle in the Colonial Ascidian *Botryllus schlosseri:* A Matter of Life and Death. Cell Tissue Res (2008) 331:555–64. 10.1007/s00441-007-0513-4 17972103

[137] Kowarsky M Anselmi C Hotta K Burighel P Zaniolo G Caicci F. Sexual and Asexual Development: Two Distinct Programs Producing the Same Tunicate. Cell Rep (2021) 34:108681. 10.1016/j.celrep.2020.108681 33503429 PMC7949349

[138] Franchi N Ballarin L. Preliminary Characterization of Complement in a Colonial Tunicate: C3, Bf and Inhibition of C3 Opsonic Activity by Compstatin. Dev Comp Immunol (2014) 46:430–8. 10.1016/j.dci.2014.05.014 24877658

[139] Peronato A Drago L Rothbächer U Macor P Ballarin L Franchi N. Complement System and Phagocytosis in a Colonial Protochordate. Dev Comp Immunol (2020) 103:103530. 10.1016/j.dci.2019.103530 31669308

[140] Voskoboynik A Rinkevich B Weiss A Moiseeva E Reznick AZ. Macrophage Involvement for Successful Degeneration of Apoptotic Organs in the Colonial Urochordate *Botryllus schlosseri* . J Exp Biol (2004) 207:2409–16. 10.1242/jeb.01045 15184513

[141] Laird DJ De Tomaso AW Weissman IL. Stem Cells Are Units of Natural Selection in a Colonial Ascidian. Cell (2005) 123:1351–60. 10.1016/j.cell.2005.10.026 16377573

[142] Burighel P Brunetti R. The Circulatory System in the Blastozooid of the Colonial Ascidian *Botryllus schlosseri* (Pallas). Ital J Zool (1971) 38:273–89. 10.1080/11250007109429158

[143] Rinkevich Y Voskoboynik A Rosner A Rabinowitz C Paz G Oren M. Repeated, Long-Term Cycling of Putative Stem Cells Between Niches in a Basal Chordate. Dev Cell (2013) 24:76–88. 10.1016/j.devcel.2012.11.010 23260626 PMC3810298

[144] Oka H Watanabe H. Vascular Budding, a New Type of Budding in. Botryllus Biol Bull (1957) 112:225–40. 10.2307/1539200

[145] Freeman G. The Role of Blood Cells in the Process of Asexual Reproduction in the Tunicate *Perophora viridis* . J Exp Zool (1964) 156:157–83. 10.1002/jez.1401560204 14193843

[146] Sabbadin A Zaniolo G Majone F. Determination of Polarity and Bilateral Asymmetry in Palleal and Vascular Buds of the Ascidian *Botryllus schlosseri* . Dev Biol (1975) 46:79–87. 10.1016/0012-1606(75)90088-3 1158028

[147] Voskoboynik A Simon-Blecher N Soen Y Rinkevich B De Tomaso AW Ishizuka KJ. Striving for Normality: Whole Body Regeneration Through a Series of Abnormal Generations. FASEB J (2007) 21:1335–44. 10.1096/fj.06-7337com 17289924

[148] Rinkevich Y Douek J Haber O Rinkevich B Reshef R. Urochordate Whole Body Regeneration Inaugurates a Diverse Innate Immune Signaling Profile. Dev Biol (2007) 312:131–46. 10.1016/j.ydbio.2007.09.005 17964563

[149] Rinkevich Y Paz G Rinkevich B Reshef R. Systemic Bud Induction and Retinoic Acid Signaling Underlie Whole Body Regeneration in the Urochordate *Botrylloides leachi* . PloS Biol (2007) 5:900–13. 10.1371/journal.pbio.0050071 PMC1808485 17341137

[150] Rinkevich Y Rinkevich B Reshef R. Cell Signaling and Transcription Factor Genes Expressed During Whole Body Regeneration in a Colonial Chordate. BMC Dev Biol (2008) 8:1–11. 10.1186/1471-213X-8-100 18847507 PMC2576188

[151] Zondag LE Rutherford K Gemmell NJ Wilson MJ. Uncovering the Pathways Underlying Whole Body Regeneration in a Chordate Model, *Botrylloides leachi* Using *De Novo* Transcriptome Analysis. BMC Genomics (2016) 17:1–11. 10.1186/s12864-016-2435-6 26879048 PMC4755014

[152] Blanchoud S Zondag L Lamare MD Wilson MJ. Hematological Analysis of the Ascidian *Botrylloides leachii* (Savigny, 1816) During Whole-Body Regeneration. Biol Bull (2017) 232:143–57. 10.1086/692841 28898595

[153] Ballarin L del Favero M Manni L. Relationships Among Hemocytes, Tunic Cells, Germ Cells, and Accessory Cells in the Colonial Ascidian *Botryllus schlosseri* . J Exp Zool Part B Mol Dev Evol (2011) 316 B:284–95. 10.1002/jez.b.21400 21246708

[154] Cloney RA. Larval Tunic and the Function of the Test Cells in Ascidians. Acta Zool (1990) 71:151–9. 10.1111/j.1463-6395.1990.tb01190.x

[155] Manni L Zaniolo G Burighel P. Egg Envelope Cytodifferentiation in the Colonial Ascidian *Botryllus schlosseri* (Tunicata). Acta Zool (1993) 74:103–13. 10.1111/j.1463-6395.1993.tb01226.x

[156] Rosner A Moiseeva E Rabinowitz C Rinkevich B. Germ Lineage Properties in the Urochordate *Botryllus schlosseri* - From Markers to Temporal Niches. Dev Biol (2013) 384:356–74. 10.1016/j.ydbio.2013.10.002 24120376

[157] Xue Q Liu H Zeng Q Zheng H Xue Q Cai X. The DEAD-Box RNA Helicase DDX1 Interacts With the Viral Protein 3D and Inhibits Foot-and-Mouth Disease Virus Replication. Virol Sin (2019) 34:610–7. 10.1007/s12250-019-00148-7 PMC6888807 31359346

[158] Mojzesz M Rakus K Chadzinska M Nakagami K Biswas G Sakai M. Cytosolic Sensors for Pathogenic Viral and Bacterial Nucleic Acids in Fish. Int J Mol Sci (2020) 21:1–33. 10.3390/ijms21197289 PMC7582293 33023222

[159] Rosner A Kravchenko O Rinkevich B. IAP Genes Partake Weighty Roles in the Astogeny and Whole Body Regeneration in the Colonial Urochordate *Botryllus schlosseri* . Dev Biol (2019) 448:320–41. 10.1016/j.ydbio.2018.10.015 30385275

[160] Samuel T Okada K Hyer M Welsh K Zapata JM Reed JC. cIAP1 Localizes to the Nuclear Compartment and Modulates the Cell Cycle. Cancer Res (2005) 65:210–8. 15665297

[161] Rosner A Paz G Rinkevich B. Divergent Roles of the DEAD-Box Protein BS-PL10, the Urochordate Homologue of Human DDX3 and DDX3Y Proteins, in Colony Astogeny and Ontogeny. Wiley Online Libr (2006) 235:1508–21. 10.1002/dvdy.20728 16518819

[162] Weissman IL Saito Y Rinkevich B. Allorecognition Histocompatibility in a Protochordate Species: Is the Relationship to MHC Somatic or Structural? Immunol Rev (1990) 113:227–41. 10.1111/j.1600-065X.1990.tb00043.x 2180808

[163] Srivastava M Simakov O Chapman J Fahey B Gauthier MEA Mitros T. The *Amphimedon queenslandica* Genome and the Evolution of Animal Complexity. Nature (2010) 466:720–6. 10.1038/nature09201 PMC3130542 20686567

[164] Grosberg RK. The Evolution of Allorecognition Specificity in Clonal Invertebrates. Q Rev Biol (1988) 63:377–412. 10.1086/416026

[165] Karakashian S Milkman R. Colony Fusion Compatibility Types in *Botryllus schlosseri* . Biol Bull (1967) 133:473.

[166] Scofield V Nagashima L. Morphology and Genetics of Rejection Reactions Between Oozooids From the Tunicate *Botryllus schlosseri* . Biol Bull (1983) 165:733–44. 10.2307/1541475 29324005

[167] Grosberg RK Quinn JF. The Genetic Control and Consequences of Kin Recognition by the Larvae of a Colonial Marine Invertebrate. Nature (1986) 322:456–9. 10.1038/322456a0

[168] Rinkevich B Porat R Goren M. Allorecognition Elements on a Urochordate Histocompatibility Locus Indicate Unprecedented Extensive Polymorphism. Proc R Soc B Biol Sci (1995) 259:319–24. 10.1098/rspb.1995.0047

[169] Dishaw LJ Litman GW. Invertebrate Allorecognition: The Origins of Histocompatibility. Curr Biol (2009) 19:R286–8. 10.1016/j.cub.2009.02.035 PMC3699862 19368870

[170] Rosengarten RD Nicotra ML. Model Systems of Invertebrate Allorecognition. Curr Biol (2011) 21:R82–92. 10.1016/j.cub.2010.11.061 21256442

[171] Voskoboynik A Newman AM Corey DM Sahoo D Pushkarev D Neff NF. Identification of a Colonial Chordate Histocompatibility Gene. Science (2013) 341:384–7. 10.1126/science.1238036 PMC3810301 23888037

[172] Ballarin L Du Pasquier L Rinkevich B Kurtz J. Evolutionary Aspects of Allorecognition. Invertebr Surviv J (2015) 12:233–6.

[173] Chadwick-Furman N Rinkevich B. A Complex Allorecognition System in a Reef-Building Coral: Delayed Responses, Reversals and Nontransitive Hierarchies. Coral Reefs (1994) 13:57–63. 10.1007/BF00426436

[174] Rinkevich B. Links Between Alloresponses and Their Genetic Background in Colonial Urochordates and Cnidarians: Evidence for the Eecognition of ‘Nonself’ as Opposed to ‘Self’. In: Stolen JS Fletcher TC Bayne CJ Secombes CJ Zelikoff JT Twerdok L Anderson DP, editors. Modulators of Immune Responses: The Evolutionary Trail. Fair Haven: SOS Publications (1996). p. 1–13.

[175] Rinkevich B. Immunological Resorption in *Botryllus Schlosseri* (Tunicata) Chimeras Is Characterized by Multilevel Hierarchial Organization of Histocompatibility Alleles. A Speculative Endeavor. Biol Bull (1993) 184:342–5. 10.2307/1542453 29300538

[176] Rinkevich B. Neglected Biological Features in Cnidarians Self-Nonself Recognition. Adv Exp Med Biol (2012) 738:46–59. 10.1007/978-1-4614-1680-7_4 22399373

[177] Rinkevich B. Allorecognition and Xenorecognition in Reef Corals: A Decade of Interactions. Hydrobiologia (2004) 530:443–50. 10.1007/s10750-004-2686-0

[178] Sawada H Morita M Iwano M. Self/Non-Self Recognition Mechanisms in Sexual Reproduction: New Insight Into the Self-Incompatibility System Shared by Flowering Plants and Hermaphroditic Animals. Biochem Biophys Res Commun (2014) 450:1142–8. 10.1016/j.bbrc.2014.05.099 24878524

[179] Gilbert OM. Histocompatibility as Adaptive Response to Discriminatory Within-Organism Conflict: A Historical Model. Am Nat (2015) 185:228–42. 10.1086/679442 25616141

[180] Tsutsui ND. Scents of Self: The Expression Component of Self/Non-Self Recognition Systems. Ann Zool Fennici (2004) 41:713–27.

[181] Stoner DS Rinkevich B Weissman IL. Heritable Germ and Somatic Cell Lineage Competitions in Chimeric Colonial Protochordates. Proc Natl Acad Sci (1999) 96:9148–53. 10.1073/pnas.96.16.9148 PMC17747 10430910

[182] Rinkevich B. Germ Cell Parasitism as an Ecological and Evolutionary Puzzle: Hitchhiking With Positively Selected Genotypes. Oikos (2002) 96:25–30. 10.1034/j.1600-0706.2002.960102.x

[183] Rinkevich B. The Colonial Urochordate *Botryllus schlosseri*: From Stem Cells and Natural Tissue Transplantation to Issues in Evolutionary Ecology. BioEssays (2002) 24:730–40. 10.1002/bies.10123 12210534

[184] Simon-Blecher N Achituv Y Rinkevich B. Protochordate Concordant Xenotransplantation Settings Reveal Outbreaks of Donor Cells and Divergent Life Span Traits. Dev Comp Immunol (2004) 28:983–91. 10.1016/j.dci.2004.04.003 15236929

[185] Frank U Bak RPM Rinkevich B. Allorecognition Responses in the Soft Coral *Parerythropodium fulvum* Fulvum From the Red Sea. J Exp Mar Bio Ecol (1996) 197:191–201. 10.1016/0022-0981(95)00153-0

[186] Frank U Oren U Loya Y Rinkevich B. Alloimmune Maturation in the Coral *Stylophora pistillata* Is Achieved Through Three Distinctive Stages, 4 Months Post-Metamorphosis. Proc R Soc B Biol Sci (1997) 264:99–104. 10.1098/rspb.1997.0015

[187] Kurtz J Franz K. Evidence for Memory in Invertebrate Immunity. Nature (2003) 425:37–8. 10.1038/425037a 12955131

[188] Rinkevich B. Quo Vadis Chimerism? Chimerism (2011) 2:1–5. 10.4161/chim.14725 21547028 PMC3084948

[189] Cadavid LF Powell AE Nicotra ML Moreno M Buss LW. An Invertebrate Histocompatibility Complex. Genetics (2004) 167:357–65. 10.1534/genetics.167.1.357 PMC1470859 15166160

[190] Nicotra ML Powell AE Rosengarten RD Moreno M Grimwood J Lakkis FG. A Hypervariable Invertebrate Allodeterminant. Curr Biol (2009) 19:583–9. 10.1016/j.cub.2009.02.040 PMC2681180 19303297

[191] Grice LF Degnan BM. Transcriptomic Profiling of the Allorecognition Response to Grafting in the Demosponge *Amphimedon queenslandica* . Mar Drugs (2017) 15:136. 10.3390/md15050136 PMC5450542 28492509

[192] Raftos DA Tait NN Briscoe DA. Allograft Rejection and Alloimmune Memory in the Solitary Urochordate, *Styela plicata* . Dev Comp Immunol (1987) 11:343–51. 10.1016/0145-305X(87)90078-4 3622886

[193] Raftos DA Tait NN Briscoe DA. Cellular Basis of Allograft Rejection in the Solitary Urochordate,* Styela plicata* . Dev Comp Immunol (1987) 11:713–25. 10.1016/0145-305X(87)90059-0 3440499

[194] Fuke M. “ Contact Reactions” Between Xenogeneic or Allogeneic Coelomic Cells of Solitary Ascidians. Biol Bull (1980) 158:304–15. 10.2307/1540857

[195] Fuke MT Numakunai T. Allogeneic Cellular Reactions Between Intra-Specific Types of a Solitary Ascidian, *Halocynthia roretzi* . Dev Comp Immunol (1982) 6:253–61. 10.1016/S0145-305X(82)80008-6 7095231

[196] Fuke M Nakamura I. Pattern of Cellular Alloreactivity of the Solitary Ascidian, *Halocynthia roretzi*, in Relation to Genetic Control. Biol Bull (1985) 169:631–7. 10.2307/1541305

[197] Ballarin L. Ascidian Cytotoxic Cells: State of the Art and Research Perspectives. Invertebr Surviv J (2012) 9:1–6.

[198] Raftos DA Cooper EL. Proliferation of Lymphocyte-Like Cells From the Solitary Tunicate, *Styela clava*, in Response to Allogeneic Stimuli. J Exp Zool (1991) 260:391–400. 10.1002/jez.1402600313 1744619

[199] Fidler AE Bacq-Labreuil A Rachmilovitz E Rinkevich B. Efficient Dispersal and Substrate Acquisition Traits in a Marine Invasive Species *Via* Transient Chimerism and Colony Mobility. PeerJ (2018) 6:e5006. 10.7717/peerj.5006 PMC6004106 29915705

[200] Sabbadin A. Le Basi Genetiche Dell Capacita Di Fusione Fra Colonie in *Botryllus schlosseri* (Ascidiacea). Atti Accad Na Lincei Rend (1962) 32:1031–5.

[201] Oka H. Colony Specificity in Compound Ascidians. The Genetic Control of Fusibility. In: Yukawa H, editor. Profiles of Japanese Science and Scientists. Tokyo: Kodanska (1970). p. 196–206.

[202] Scofield VL Schlumpberger JM West LA Weissman IL. Protochordate Allorecognition Is Controlled by a MHC-Like Gene System. Nature (1982) 295:499–502. 10.1038/295499a0 7057909

[203] Rinkevich B Weissman IL. Allogeneic Resorption in Colonial Protochordates: Consequences of Nonself Recognition. Dev Comp Immunol (1992) 16:275–86. 10.1016/0145-305X(92)90002-T 1505688

[204] Rinkevich B. Rejection Patterns in Botryllid Ascidian Immunity: The First Tier of Allorecognition. Can J Zool (2005) 83:101–21. 10.1139/z04-161

[205] Ballarin L Cima F Sabbadin A. Morula Cells and Histocompatibility in the Colonial Ascidian *Botryllus schlosseri* . Zoolog Sci (1995) 12:757–64. 10.2108/zsj.12.757

[206] Cima F Sabbadin A Ballarin L. Cellular Aspects of Allorecognition in the Compound Ascidian *Botryllus schlosseri* . Dev Comp Immunol (2004) 28:881–9. 10.1016/j.dci.2004.02.001 15183029

[207] Rinkevich B Tartakover S Gershon H. Contribution of Morula Cells to Allogeneic Responses in the Colonial Urochordate *Botryllus schlosseri* . Mar Biol (1998) 131:227–36. 10.1007/s002270050315

[208] Ballarin L Cima F Floreani M Sabbadin A. Oxidative Stress Induces Cytotoxicity During Rejection Reaction in the Compound Ascidian *Botryllus schlosseri* . Comp Biochem Physiol C Toxicol Pharmacol (2002) 133:411–8. 10.1016/S1532-0456(02)00123-0 12379425

[209] Franchi N Ballarin L Cima F. Insights on Cytotoxic Cells of the Colonial Ascidian *Botryllus schlosseri* . Invertebr Surviv J (2015) 12:109–17.

[210] Harp JA Tsuchida CB Weissman IL Scofield VL. Autoreactive Blood Cells and Programmed Cell Death in Growth and Development of Protochordates. J Exp Zool (1988) 247:257–62. 10.1002/jez.1402470309 3183596

[211] Taneda Y Watanabe H. Effects of X-irradiation on Colony Specificity in the Compound Ascidian, *Botryllus primigenus* Oka. Dev Comp Immunol (1982) 6:665–73. 10.1016/S0145-305X(82)80007-4 7160511

[212] Rinkevich B Weissman IL. A Long-Term Study on Fused Subclones in the Ascidian *Botryllus schlosseri*: The Resorption Phenomenon (Protochordata: Tunicata). J Zool (1987) 213:717–33. 10.1111/j.1469-7998.1987.tb03736.x

[213] Rinkevich B Weissman I. The Fate of *Botryllus* (Ascidiacea) Larvae Cosettled With Parental Colonies: Beneficial or Deleterious Consequences? Biol Bull (1987) 173:474–88. 10.2307/1541694 29320221

[214] Rinkevich B Weissman IL. *Botryllus schlosseri* (Tunicata) Whole Colony Irradiation: Do Senescent Zooid Resorption and Immunological Resorption Involve Similar Recognition Events? J Exp Zool (1990) 253:189–201. 10.1002/jez.1402530209 2313247

[215] Corey DM Rosental B Kowarsky M Sinha R Ishizuka KJ Palmeri KJ. Developmental Cell Death Programs License Cytotoxic Cells to Eliminate Histocompatible Partners. Proc Natl Acad Sci (2016) 113:6520–5. 10.1073/pnas.1606276113 PMC4988592 27217570

[216] Sabbadin A Zaniolo G. Sexual Differentiation and Germ Cell Transfer in the Colonial Ascidian *Botryllus schlosseri* . J Exp Zool (1979) 207:289–304. 10.1002/jez.1402070212

[217] Sabbadin A Astorri C. Chimeras and Histocompatibility in the Colonial Ascidian *Botryllus schlosseri* . Dev Comp Immunol (1988) 12:737–47. 10.1016/0145-305X(88)90049-3 3208958

[218] Rinkevich B Weissman IL De Tomaso AW. Transplantation of Fu/HC-incompatible Zooids in *Botryllus schlosseri* Results in Chimerism. Biol Bull (1998) 195:98–106. 10.2307/1542816 9818360

[219] Rinkevich B Yankelevich I. Environmental Split Between Germ Cell Parasitism and Somatic Cell Synergism in Chimeras of a Colonial Urochordate. J Exp Biol (2004) 207:3531–6. 10.1242/jeb.01184 15339949

[220] Pancer Z Gershon H Rinkevich B. Coexistence and Possible Parasitism of Somatic and Germ Cell Lines in Chimeras of the Colonial Urochordate *Botryllus schlosseri* . Biol Bull (1995) 189:106–12. 10.2307/1542460 27768485

[221] Stoner DS Weissman IL. Somatic and Germ Cell Parasitism in a Colonial Ascidian: Possible Role for a Highly Polymorphic Allorecognition System. Proc Natl Acad Sci (1996) 93:15254–9. 10.1073/pnas.93.26.15254 PMC26390 8986797

[222] Canesi L Ciacci C Fabbri R Balbi T Salis A Damonte G. Interactions of Cationic Polystyrene Nanoparticles With Marine Bivalve Hemocytes in a Physiological Environment: Role of Soluble Hemolymph Proteins. Environ Res (2016) 150:73–81. 10.1016/j.envres.2016.05.045 27257827

[223] Canesi L Balbi T Fabbri R Salis A Damonte G Volland M. Biomolecular Coronas in Invertebrate Species: Implications in the Environmental Impact of Nanoparticles. NanoImpact (2017) 8:89–98. 10.1016/j.impact.2017.08.001

[224] Ambrosone A Mattera L Marchesano V Quarta A Susha AS Tino A. Mechanisms Underlying Toxicity Induced by CdTe Quantum Dots Determined in an Invertebrate Model Organism. Biomaterials (2012) 33:1991–2000. 10.1016/j.biomaterials.2011.11.041 22169823

[225] Xing R Li K-L Zhou Y-F Su Y-Y Yan S-Q Zhang K-L. Impact of Fluorescent Silicon Nanoparticles on Circulating Hemolymph and Hematopoiesis in an Invertebrate Model Organism. Chemosphere (2016) 159:628–37. 10.1016/j.chemosphere.2016.06.057 27348562

[226] Fang Y He Y Wang H Yan S Xing R Yin W. Hematopoiesis Toxicity Induced by 4-Methylumbelliferon Determined in an Invertebrate Model Organism. Drug Chem Toxicol (2016) 39:199–205. 10.3109/01480545.2015.1079915 26327572

[227] Liu T Xing R Zhou YF Zhang J Su YY Zhang KQ. Hematopoiesis Toxicity Induced by CdTe Quantum Dots Determined in an Invertebrate Model Organism. Biomaterials (2014) 35:2942–51. 10.1016/j.biomaterials.2013.12.007 24411333

[228] Tan J Xu M Zhang K Wang X Chen S Li T. Characterization of Hemocytes Proliferation in Larval Silkworm *Bombyx mori* . J Insect Physiol (2013) 59:595–603. 10.1016/j.jinsphys.2013.03.008 23557681

[229] Oweson C Sköld H Pinsino A Matranga V Hernroth B. Manganese Effects on Haematopoietic Cells and Circulating Coelomocytes of. Asterias Rubens (Linnaeus) Aquat Toxicol (2008) 89:75–81. 10.1016/j.aquatox.2008.05.016 18639346

[230] Betti M Canonico B Ciacci C Lorusso LC Papa S Canesi L. Effects of Stem Cell Factor (SCF) on Proliferation and Differentiation of Bivalve Immunocytes. Comp Biochem Physiol Part A Mol Integr Physiol (2008) 151:S44–5. 10.1016/j.cbpa.2008.05.129

[231] Aladaileh S Nair SV Birch D Raftos DA. Sydney Rock Oyster (*Saccostrea Glomerata*) Hemocytes: Morphology and Function. J Invertebr Pathol (2007) 96:48–63. 10.1016/j.jip.2007.02.011 17412360

[232] Dolar A Kostanjšek R Mayall C Drobne D Kokalj AJ. Modulations of Immune Parameters Caused by Bacterial and Viral Infections in the Terrestrial Crustacean *Porcellio scaber*: Implications for Potential Markers in Environmental Research. Dev Comp Immunol (2020) 113:103789. 10.1016/j.dci.2020.103789 32735963

[233] Cima F Perin A Burighel P Ballarin L. Morpho-Functional Characterization of Haemocytes of the Compound Ascidian *Botrylloides leachi* (Tunicata, Ascidiacea). Acta Zool (2001) 82:261–74. 10.1046/j.1463-6395.2001.00087.x

[234] Nguyen PD Currie PD. *In Vivo* Imaging: Shining a Light on Stem Cells in the Living Animal. Development (2018) 145dev150441. 10.1242/dev.150441 29592949

[235] Lv S Xu J Zhao J Yin N Lu B Li S. Classification and Phagocytosis of Circulating Haemocytes in Chinese Mitten Crab (*Eriocheir Sinensis*) and the Effect of Extrinsic Stimulation on Circulating Haemocytes Invivo. Fish shellfish Immunol (2014) 39:415–22. 10.1016/j.fsi.2014.05.036 24929244

[236] Zhou YL Gu W Bin Tu DD Zhu QH Zhou ZK. Hemocytes of the Mud Crab *Scylla paramamosain*: Cytometric, Morphological Characterization and Involvement in Immune Responses. Fish shellfish Immunol (2018) 72:459–69. 10.1016/j.fsi.2017.10.055 29108971

[237] Castellanos-Martínez S Prado-Alvarez M Lobo-da-Cunha A Azevedo C Gestal C. Morphologic, Cytometric and Functional Characterization of the Common Octopus (*Octopus vulgaris*) Hemocytes. Dev Comp Immunol (2014) 44:50–8. 10.1016/j.dci.2013.11.013 24296436

[238] Odintsova NA. Stem Cells of Marine Invertebrates: Regulation of Proliferation and Induction of Differentiation *In Vitro* . Cell Tissue Biol (2009) 3:403–8. 10.1134/S1990519X09050010 19505055

[239] Bosch TCG. Hydra and the Evolution of Stem Cells. BioEssays (2009) 31:478–86. 10.1002/bies.200800183 19274660

[240] Holland ND Somorjai IML. Serial Blockface SEM Suggests That Stem Cells may Participate in Adult Notochord Growth in an Invertebrate Chordate, the Bahamas Lancelet. Evodevo (2020) 11:22. 10.1186/s13227-020-00167-6 33088474 PMC7568382

[241] Zhang ZF Shao M Ho Kang K. Classification of Haematopoietic Cells and Haemocytes in Chinese Prawn *Fenneropenaeus chinensis* . Fish Shellfish Immunol (2006) 21:159–69. 10.1016/j.fsi.2005.11.003 16480894

[242] Giulianini PG Bierti M Lorenzon S Battistella S Ferrero EA. Ultrastructural and Functional Characterization of Circulating Hemocytes From the Freshwater Crayfish *Astacus leptodactylus*: Cell Types and Their Role After *In Vivo* Artificial Non-Self Challenge. Micron (2007) 38:49–57. 10.1016/j.micron.2006.03.019 16839768

[243] Chevalier F Herbinière-Gaboreau J Bertaux J Raimond M Morel F Bouchon D. The Immune Cellular Effectors of Terrestrial Isopod *Armadillidium vulgare*: Meeting With Their Invaders, Wolbachia. PloS One (2011) 6:e18531. 10.1371/journal.pone.0018531 21533137 PMC3080368

[244] Rebelo M de F Figueiredo E de S Mariante RM Nóbrega A de Barros CM Allodi S. New Insights From the Oyster *Crassostrea rhizophorae* on Bivalve Circulating Hemocytes. PloS One (2013) 8:e57384. 10.1371/journal.pone.0057384 23451217 PMC3581465

[245] Mangkalanan S Sanguanrat P Utairangsri T Sritunyalucksana K Krittanai C. Characterization of the Circulating Hemocytes in Mud Crab (*Scylla olivacea*) Revealed Phenoloxidase Activity. Dev Comp Immunol (2014) 44:116–23. 10.1016/j.dci.2013.11.018 24316230

[246] Tame A Yoshida T Ohishi K Maruyama T. Phagocytic Activities of Hemocytes From the Deep-Sea Symbiotic Mussels *Bathymodiolus Japonicus*, *B. platifrons* and* B. septemdierum* . Fish Shellfish Immunol (2015) 45:146–56. 10.1016/j.fsi.2015.03.020 25804489

[247] Preziosi BM Bowden TJ. Morphological Characterization *Via* Light and Electron Microscopy of Atlantic Jackknife Clam (*Ensis directus*) Hemocytes. Micron (2016) 84:96–106. 10.1016/j.micron.2016.03.003 27015289

[248] Thayappan K Denis M Mullaivanam Ramasamy S Munusamy A. Hemocytes and Hemocytic Responses in the Mole Crab *Emerita emeritus* (Linnaeus 1767). J Invertebr Pathol (2017) 148:129–37. 10.1016/j.jip.2017.06.011 28668255

[249] Wu F Xie Z Yan M Li Q Song J Hu M. Classification and Characterization of Hemocytes From Two Asian Horseshoe Crab Species *Tachypleus tridentatus* and *Carcinoscorpius rotundicauda* . Sci Rep (2019) 9:1–10. 10.1038/s41598-019-43630-8 31068640 PMC6506590

[250] Hose JE Martin GG Gerard AS. A Decapod Hemocyte Classification Scheme Integrating Morphology, Cytochemistry, and Function. Biol Bull (1990) 178:33–45. 10.2307/1541535 29314975

[251] Higuchi S Hayashi T Hori I Shibata N Sakamoto H Agata K. Characterization and Categorization of Fluorescence Activated Cell Sorted Planarian Stem Cells by Ultrastructural Analysis. Dev Growth Differ (2007) 49:571–81. 10.1111/j.1440-169x.2007.00947.x 17587325

[252] Söderhäll I. Crustacean Hematopoiesis. Dev Comp Immunol (2016) 58:129–41. 10.1016/j.dci.2015.12.009 26721583

[253] Wu C Söderhäll I Kim YA Liu H Söderhäll K. Hemocyte-Lineage Marker Proteins in a Crustacean, the Freshwater Crayfish *Pacifastacus leniusculus* . Proteomics (2008) 8:4226–35. 10.1002/pmic.200800177 18814328

[254] Wu C Charoensapsri W Nakamura S Tassanakajon A Söderhäll I Söderhäll K. An MBL-Like Protein may Interfere With the Activation of the proPO-system, an Important Innate Immune Reaction in Invertebrates. Immunobiology (2013) 218:159–68. 10.1016/j.imbio.2012.02.011 22459272

[255] Mochizuki K Nishimiya-Fujisawa C Fujisawa T. Universal Occurrence of the Vasa-Related Genes Among Metazoans and Their Germline Expression in *Hydra* . Dev Genes Evol (2001) 211:299–308. 10.1007/s004270100156 11466525

[256] Önal P Grün D Adamidi C Rybak A Solana J Mastrobuoni G. Gene Expression of Pluripotency Determinants Is Conserved Between Mammalian and Planarian Stem Cells. EMBO J (2012) 31:2755–69. 10.1038/emboj.2012.110 PMC3380209 22543868

[257] Fierro-Constaín L Schenkelaars Q Gazave E Haguenauer A Rocher C Ereskovsky A. The Conservation of the Germline Multipotency Program, From Sponges to Vertebrates: A Stepping Stone to Understanding the Somatic and Germline Origins. Genome Biol Evol (2017) 9:evw289. 10.1093/gbe/evw289 PMC5381599 28082608

[258] Cao C Lemaire LA Wang W Yoon PH Choi YA Parsons LR. Comprehensive Single-Cell Transcriptome Lineages of a Proto-Vertebrate. Nature (2019) 571:349–54. 10.1038/s41586-019-1385-y PMC6978789 31292549

[259] Reddien PW Oviedo NJ Jennings JR Jenkin JC Sánchez Alvarado A. Developmental Biology: SMEDWI-2 Is a PIWI-Like Protein That Regulates Planarian Stem Cells. Science (2005) 310:1327–30. 10.1126/science.1116110 16311336

[260] Pfister D De Mulder K Philipp I Kuales G Hrouda M Eichberger P. The Exceptional Stem Cell System of *Macrostomum lignano*: Screening for Gene Expression and Studying Cell Proliferation by Hydroxyurea Treatment and Irradiation. Front Zool (2007) 4:1–14. 10.1186/1742-9994-4-9 17349046 PMC1828727

[261] Palakodeti D Smielewska M Lu YC Yeo GW Graveley BR. The PIWI Proteins SMEDWI-2 and SMEDWI-3 Are Required for Stem Cell Function and piRNA Expression in Planarians. RNA (2008) 14:1174–86. 10.1261/rna.1085008 PMC2390803 18456843

[262] Rinkevich Y Rosner A Rabinowitz C Lapidot Z Moiseeva E Rinkevich B. Piwi Positive Cells That Line the Vasculature Epithelium, Underlie Whole Body Regeneration in a Basal Chordate. Dev Biol (2010) 345:94–104. 10.1016/j.ydbio.2010.05.500 20553710

[263] Alié A Leclère L Jager M Dayraud C Chang P Le Guyader H. Somatic Stem Cells Express Piwi and Vasa Genes in an Adult Ctenophore: Ancient Association of “Germline Genes” With Stemness. Dev Biol (2011) 350:183–97. 10.1016/j.ydbio.2010.10.019 21036163

[264] Hobmayer B Jenewein M Eder D Eder M-K Glasauer S Gufler S. Stemness in *Hydra* - A Current Perspective. Int J Dev Biol (2012) 56:509–17. 10.1387/ijdb.113426bh 22689357

[265] Gazave E Béhague J Laplane L Guillou A Préau L Demilly A. Posterior Elongation in the Annelid *Platynereis dumerilii* Involves Stem Cells Molecularly Related to Primordial Germ Cells. Dev Biol (2013) 382:246–67. 10.1016/j.ydbio.2013.07.013 23891818

[266] Rosental B Kowarsky M Seita J Corey DM Ishizuka KJ Palmeri KJ. Complex Mammalian-Like Haematopoietic System Found in a Colonial Chordate. Nature (2018) 564:425–9. 10.1038/s41586-018-0783-x PMC6347970 30518860

[267] Rinkevich B. Cell Cultures From Marine Invertebrates: Obstacles, New Approaches and Recent Improvements. J Biotechnol (1999) 70:133–53. 10.1016/S0168-1656(99)00067-X

[268] Rinkevich B. Cell Cultures From Marine Invertebrates: New Insights for Capturing Endless Stemness. Mar Biotechnol (2011) 13:345–54. 10.1007/s10126-010-9354-3 21213116

[269] Thammasorn T Nozaki R Kondo H Hirono I. Investigation of Essential Cell Cycle Regulator Genes as Candidates for Immortalized Shrimp Cell Line Establishment Based on the Effect of *In Vitro* Culturing on Gene Expression of Shrimp Primary Cells. Aquaculture (2020) 529:735733. 10.1016/j.aquaculture.2020.735733

[270] Conkling M Hesp K Munroe S Sandoval K Martens DE Sipkema D. Breakthrough in Marine Iunvertebrate Cell Culture: Sponge Cells Divide Rapidly in Improved Nutrient Medium. Sci Rep (2019) 9:1–10. 10.1038/s41598-019-53643-y 31754216 PMC6872747

[271] Munroe S Sandoval K Martens DE Sipkema D Pomponi SA. Genetic Algorithm as an Optimization Tool for the Development of Sponge Cell Culture Media. Vitr Cell Dev Biol Anim (2019) 55:149–58. 10.1007/s11626-018-00317-0 PMC6407725 30747414

[272] Revilla-i-Domingo R Schmidt C Zifko C Raible F. Establishment of Transgenesis in the Demosponge *Suberites domuncula* . Genetics (2018) 210:435–43. 10.1534/genetics.118.301121 PMC6216596 30143594

[273] Ventura P Toullec G Fricano C Chapron L Meunier V Röttinger E. Cnidarian Primary Cell Culture as a Tool to Investigate the Effect of Thermal Stress at Cellular Level. Mar Biotechnol (2018) 20:144–54. 10.1007/s10126-017-9791-3 29313151

[274] Söderhäll I Kim Y-A Jiravanichpaisal P Lee S-Y Söderhäll K. An Ancient Role for a Prokineticin Domain in Invertebrate Hematopoiesis. J Immunol (2005) 174:6153–60. 10.4049/jimmunol.174.10.6153 15879111

[275] Xu X Duan H Shi Y Xie S Song Z Jin S. Development of a Primary Culture System for Haematopoietic Tissue Cells From *Cherax quadricarinatus* and an Exploration of Transfection Methods. Dev Comp Immunol (2018) 88:45–54. 10.1016/j.dci.2018.07.006 30003889

[276] Morgan SR Paletto L Rumney B Malik FT White N Lewis PN. Establishment of Long-Term Ostracod Epidermal Culture. Vitr Cell Dev Biol Anim (2020) 56:760–72. 10.1007/s11626-020-00508-8 PMC7658072 33034828

[277] Pinsino A Alijagic A. Sea Urchin *Paracentrotus lividus* Immune Cells in Culture: Formulation of the Appropriate Harvesting and Culture Media and Maintenance Conditions. Biol Open (2019) 8bio039289. 10.1242/bio.039289 PMC6451355 30718227

[278] Dehal P Satou Y Campbell RK Chapman J Degnan B De Tomaso A. The Draft Genome of *Ciona intestinalis*: Insights Into Chordate and Vertebrate Origins. Science (2002) 298:2157–67. 10.1126/science.1080049 12481130

[279] Södergren E Weinstock GM Davidson EH Cameron RA Gibbs R. The Genome of the Sea Urchin *Strongylocentrotus purpuratus* . Science (2006) 314:941–52. 10.1126/science.1133609 PMC3159423 17095691

[280] Small KS Brudno M Hill MM Sidow A. A Haplome Alignment and Reference Sequence of the Highly Polymorphic *Ciona savignyi* Genome. Genome Biol (2007) 8:R41. 10.1186/gb-2007-8-3-r41 17374142 PMC1868934

[281] Srivastava M Begovic E Chapman J Putnam NH Hellsten U Kawashima T. The Trichoplax Genome and the Nature of Placozoans. Nature (2008) 454:955–60. 10.1038/nature07191 18719581

[282] Ryan JF Pang K Mullikin JC Martindale MQ Baxevanis AD. The Homeodomain Complement of the Ctenophore *Mnemiopsis leidyi* Suggests That Ctenophora and Porifera Diverged Prior to the ParaHoxozoa. Evodevo (2010) 1:1–18. 10.1186/2041-9139-1-9 20920347 PMC2959044

[283] Nichols SA Roberts BW Richter DJ Fairclough SR King N. Origin of Metazoan Cadherin Diversity and the Antiquity of the Classical Cadherin/β-Catenin Complex. Proc Natl Acad Sci (2012) 109:13046–51. 10.1073/pnas.1120685109 PMC3420208 22837400

[284] Voskoboynik A Neff NF Sahoo D Newman AM Pushkarev D Koh W. The Genome Sequence of the Colonial Chordate. Botryllus schlosseri Elife (2013) 2:e00569. 10.7554/eLife.00569 23840927 PMC3699833

[285] Fortunato SAV Adamski M Ramos OM Leininger S Liu J Ferrier DEK. Calcisponges Have a ParaHox Gene and Dynamic Expression of Dispersed NK Homeobox Genes. Nature (2014) 514:620–3. 10.1038/nature13881 25355364

[286] Stolfi A Lowe EK Racioppi C Ristoratore F Brown CT Swalla BJ. Divergent Mechanisms Regulate Conserved Cardiopharyngeal Development and Gene Expression in Distantly Related Ascidians. Elife (2014) 3:e03728. 10.7554/eLife.03728 25209999 PMC4356046

[287] Brozovic M Martin C Dantec C Dauga D Mendez M Simion P. Aniseed 2015: A Digital Framework for the Comparative Developmental Biology of Ascidians. Nucleic Acids Res (2016) 44:D808–18. 10.1093/nar/gkv966 PMC4702943 26420834

[288] Ereskovsky AV Richter DJ Lavrov DV Schippers KJ Nichols SA. Transcriptome Sequencing and Delimitation of Sympatric *Oscarella* Species (*O. carmela* and *O. pearsei* Sp. Nov) From California, USA. PloS One (2017) 12:e0183002. 10.1371/journal.pone.0183002 28892487 PMC5593202

[289] Francis WR Eitel M Vargas S Adamski M Haddock SHD Krebs S. The Genome of the Contractile Demosponge *Tethya wilhelma* and the Evolution of Metazoan Neural Signalling Pathways. bioRxiv (2017) 120998. 10.1101/120998. bioRxiv.

[290] Gomes-dos-Santos A Lopes-Lima M Castro LFC Froufe E. Molluscan Genomics: The Road So Far and the Way Forward. Hydrobiologia (2020) 847:1705–26. 10.1007/s10750-019-04111-1

[291] Hamada M Satoh N Khalturin K. A Reference Genome From the Symbiotic Hydrozoan, *Hydra viridissima* . G3 Genes Genomes Genet (2020) 10:3883–95. 10.1534/g3.120.401411 PMC7642931 32900905

[292] Magie CR Pang K Martindale MQ. Genomic Inventory and Expression of Sox and Fox Genes in the Cnidarian *Nematostella vectensis* . Dev Genes Evol (2005) 215:618–30. 10.1007/s00427-005-0022-y 16193320

[293] Vinson JP Jaffe DB O’Neill K Karlsson EK Stange-Thomann N Anderson S. Assembly of Polymorphic Genomes: Algorithms and Application to *Ciona savignyi* . Genome Res (2005) 15:1127–35. 10.1101/gr.3722605 PMC1182225 16077012

[294] Gómez-Chiarri M Warren WC Guo X Proestou D. Developing Tools for the Study of Molluscan Immunity: Thesequencing of the Genome of the Eastern Oyster, *Crassostrea virginica* . Fish Shellfish Immunol (2015) 46:2–4. 10.1016/j.fsi.2015.05.004 25982405

[295] Gerdol M Moreira R Cruz F Gómez-Garrido J Vlasova A Rosani U. Massive Gene Presence-Absence Variation Shapes an Open Pan-Genome in the Mediterranean Mussel. Genome Biol (2020) 21:275. 10.1186/s13059-020-02180-3 33168033 PMC7653742

[296] Mao F Wong N-K Lin Y Zhang X Liu K Huang M. Transcriptomic Evidence Reveals the Molecular Basis for Functional Differentiation of Hemocytes in a Marine Invertebrate, *Crassostrea gigas* . Front Immunol (2020) 11:911. 10.3389/fimmu.2020.00911 32536915 PMC7269103

[297] Söderhäll I Junkunlo K. A Comparative Global Proteomic Analysis of the Hematopoietic Lineages in the Crustacean *Pacifastacus leniusculus* . Dev Comp Immunol (2019) 92:170–8. 10.1016/j.dci.2018.11.016 30481524

[298] Angajala A Lim S Phillips JB Kim JH Yates C You Z. Diverse Roles of Mitochondria in Immune Responses: Novel Insights Into Immuno-Metabolism. Front Immunol (2018) 9:1605. 10.3389/fimmu.2018.01605 30050539 PMC6052888

[299] Zhou R Yazdi AS Menu P Tschopp J. A Role for Mitochondria in NLRP3 Inflammasome Activation. Nature (2011) 469:221–6. 10.1038/nature09663 21124315

[300] Grimm S. The ER-Mitochondria Interface: The Social Network of Cell Death. Biochim Biophys Acta Mol Cell Res (2012) 1823:327–34. 10.1016/j.bbamcr.2011.11.018 22182703

[301] Paliwal S Fiumera HL Mohanty S. Stem Cell Plasticity and Regenerative Potential Regulation Through Ca2+-Mediated Mitochondrial Nuclear Crosstalk. Mitochondrion (2021) 56:1–14. 10.1016/j.mito.2020.10.002 33059088

[302] Bernitz JM Kim HS MacArthur B Sieburg H Moore K. Hematopoietic Stem Cells Count and Remember Self-Renewal Divisions. Cell (2016) 167:1296–309. 10.1016/j.cell.2016.10.022 PMC5115957 27839867

[303] Hinge A He J Bartram J Javier J Xu J Fjellman E. Asymmetrically Segregated Mitochondria Provide Cellular Memory of Hematopoietic Stem Cell Replicative History and Drive HSC Attrition. Cell Stem Cell (2020) 26:420–30. 10.1016/j.stem.2020.01.016 PMC7212526 32059807

[304] Rastogi A Joshi P Contreras E Gama V. Remodeling of Mitochondrial Morphology and Function: An Emerging Hallmark of Cellular Reprogramming. Cell Stress (2019) 3:181–94. 10.15698/cst2019.06.189 PMC6558935 31225513

[305] Xu X Duan S Yi F Ocampo A Liu GH Izpisua Belmonte JC. Mitochondrial Regulation in Pluripotent Stem Cells. Cell Metab (2013) 18:325–32. 10.1016/j.cmet.2013.06.005 23850316

[306] Stubbington MJT Rozenblatt-Rosen O Regev A Teichmann SA. Single-Cell Transcriptomics to Explore the Immune System in Health and Disease. Science (2017) 358:58–63. 10.1126/science.aan6828 28983043 PMC5654495

[307] Briggs JA Weinreb C Wagner DE Megason S Peshkin L Kirschner MW. The Dynamics of Gene Expression in Vertebrate Embryogenesis at Single-Cell Resolution. Science (2018) 360:eaar5780. 10.1126/science.aar5780 29700227 PMC6038144

[308] Farrell JA Wang Y Riesenfeld SJ Shekhar K Regev A Schier AF. Single-Cell Reconstruction of Developmental Trajectories During Zebrafish Embryogenesis. Science (2018) 360:6392. 10.1126/science.aar3131 PMC6247916 29700225

[309] Sebé-Pedrós A Saudemont B Chomsky E Plessier F Mailhé MP Renno J. Cnidarian Cell Type Diversity and Regulation Revealed by Whole-Organism Single-Cell RNA-Seq. Cell (2018) 173:1520–34. 10.1016/j.cell.2018.05.019 29856957

[310] Sebé-Pedrós A Chomsky E Pang K Lara-Astiaso D Gaiti F Mukamel Z. Early Metazoan Cell Type Diversity and the Evolution of Multicellular Gene Regulation. Nat Ecol Evol (2018) 2:1176–88. 10.1038/s41559-018-0575-6 PMC6040636 29942020

[311] Zhong S Zhang S Fan X Wu Q Yan L Dong J. A Single-Cell RNA-seq Survey of the Developmental Landscape of the Human Prefrontal Cortex. Nature (2018) 555:524–8. 10.1038/nature25980 29539641

[312] Wagner DE Weinreb C Collins ZM Briggs JA Megason SG Klein AM. Single-Cell Mapping of Gene Expression Landscapes and Lineage in the Zebrafish Embryo. Science (2018) 360:981–7. 10.1126/science.aar4362 PMC6083445 29700229

[313] Cao J Spielmann M Qiu X Huang X Ibrahim DM Hill AJ. The Single-Cell Transcriptional Landscape of Mammalian Organogenesis. Nature (2019) 566:496–502. 10.1038/s41586-019-0969-x 30787437 PMC6434952

[314] Musser JM Schippers KJ Nickel M Mizzon G Kohn AB Pape C. Profiling Cellular Diversity in Sponges Informs Animal Cell Type and Nervous System Evolution. bioRxiv (2019) 758276. 10.1101/758276 PMC9233960 34735222

[315] Pijuan-Sala B Griffiths JA Guibentif C Hiscock TW Jawaid W Calero-Nieto FJ. A Single-Cell Molecular Map of Mouse Gastrulation and Early Organogenesis. Nature (2019) 566:490–5. 10.1038/s41586-019-0933-9 PMC6522369 30787436

[316] Sharma S Wang W Stolfi A. Single-Cell Transcriptome Profiling of the *Ciona* Larval Brain. Dev Biol (2019) 448:226–36. 10.1016/j.ydbio.2018.09.023 PMC6487232 30392840

[317] Fincher CT Wurtzel O de Hoog T Kravarik KM Reddien PW. Cell Type Transcriptome Atlas for the Planarian *Schmidtea mediterranea* . Science (2018) 360:6391. 10.1126/science.aaq1736 PMC6563842 29674431

[318] Plass M Solana J Alexander Wolf F Ayoub S Misios A. Glažar P Cell Type Atlas and Lineage Tree of a Whole Complex Animal by Single-Cell Transcriptomics. Science (2018) 360:6391. 10.1126/science.aaq1723 29674432

[319] Zhou Y Kim J Yuan X Braun T. Epigenetic Modifications of Stem Cells: A Paradigm for the Control of Cardiac Progenitor Cells. Circ Res (2011) 109:1067–81. 10.1161/CIRCRESAHA.111.243709 21998298

[320] Fellous A Lefranc L Jouaux A Goux D Favrel P Rivière G. Histone Methylation Participates in Gene Expression Control During the Early Development of the Pacific Oyster *Crassostrea gigas* . Genes (2019) 10:695. 10.3390/genes10090695 PMC6771004 31509985

[321] Chen YP Yin JH Li WF Li HJ Chen DP Zhang CJ. Single-Cell Transcriptomics Reveals Regulators Underlying Immune Cell Diversity and Immune Subtypes Associated With Prognosis in Nasopharyngeal Carcinoma. Cell Res (2020) 30:1024–42. 10.1038/s41422-020-0374-x PMC7784929 32686767

[322] Deaton AM Bird A. Cpg Islands and the Regulation of Transcription. Genes Dev (2011) 25:1010–22. 10.1101/gad.2037511 PMC3093116 21576262

[323] Dattani A Kao D Mihaylova Y Abnave P Hughes S Lai A. Epigenetic Analyses of Planarian Stem Cells Demonstrate Conservation of Bivalent Histone Modifications in Animal Stem Cells. Genome Res (2018) 28:1543–54. 10.1101/gr.239848.118 PMC6169894 30143598

[324] Shibuya M Hatano M Kawamura K. Interactive Histone Acetylation and Methylation in Regulating Transdifferentiation-Related Genes During Tunicate Budding and Regeneration. Dev Dyn (2015) 244:10–20. 10.1002/dvdy.24212 25298085

[325] Park PJ. Chip-Seq: Advantages and Challenges of a Maturing Technology. Nat Rev Genet (2009) 10:669–80. 10.1038/nrg2641 PMC3191340 19736561

[326] Birney E Stamatoyannopoulos JA Dutta A Guigó R Gingeras TR Margulies EH. Identification and Analysis of Functional Elements in 1% of the Human Genome by the ENCODE Pilot Project. Nature (2007) 447:799–816. 10.1038/nature05874 17571346 PMC2212820

[327] Celniker SE Dillon LAL Gerstein MB Gunsalus KC Henikoff S Karpen GH. Unlocking the Secrets of the Genome. Nature (2009) 459:927–30. 10.1038/459927a PMC2843545 19536255

[328] Dobber R Hertogh-Huijbregts A Rozing J Nagelkerken L Bottomly K. The Involvement of the Intestinal Microflora in the Expansion of Cd4+ T Cells With a Naive Phenotype in the Periphery. Dev Immunol (1992) 2:141–50. 10.1155/1992/57057 PMC2275855 1386544

[329] Geuking MB Köller Y Rupp S McCoy KD. The Interplay Between the Gut Microbiota and the Immune System. Gut Microbes (2014) 5:411–8. 10.4161/gmic.29330 PMC4153781 24922519

[330] Gensollen T Iyer SS Kasper DL Blumberg RS. How Colonization by Microbiota in Early Life Shapes the Immune System. Science (2016) 352:539–44. 10.1126/science.aad9378 PMC5050524 27126036

[331] Negi S Pahari S Bashir H Agrewala JN. Gut Microbiota Regulates Mincle Mediated Activation of Lung Dendritic Cells to Protect Against *Mycobacterium tuberculosis* . Front Immunol (2019) 10:1142. 10.3389/fimmu.2019.01142 31231363 PMC6558411

[332] Franzenburg S Walter J Künzel S Wang J Baines JF Bosch TCG. Distinct Antimicrobial Peptide Expression Determines Host Species-Specific Bacterial Associations. Proc Natl Acad Sci USA (2013) 110:E3730–8. 10.1073/pnas.1304960110 PMC3785777 24003149

[333] Fakhoury M Negrulj R Mooranian A Al-Salami H. Inflammatory Bowel Disease: Clinical Aspects and Treatments. J Inflammation Res (2014) 7:113–20. 10.2147/JIR.S65979 PMC4106026 25075198

[334] Ocansey DKW Wang L Wang J Yan Y Qian H Zhang X. Mesenchymal Stem Cell–Gut Microbiota Interaction in the Repair of Inflammatory Bowel Disease: An Enhanced Therapeutic Effect. Clin Transl Med (2019) 8:1–17. 10.1186/s40169-019-0251-8 31872304 PMC6928179

[335] Rodrigues J Brayner FA Alves LC Dixit R Barillas-Mury C. Hemocyte Differentiation Mediates Innate Immune Memory in *Anopheles gambiae* Mosquitoes. Science (2010) 329:1353–5. 10.1126/science.1190689 PMC3510677 20829487

[336] Ganal SC Sanos SL Kallfass C Oberle K Johner C Kirschning C. Priming of Natural Killer Cells by Nonmucosal Mononuclear Phagocytes Requires Instructive Signals From Commensal Microbiota. Immunity (2012) 37:171–86. 10.1016/j.immuni.2012.05.020 22749822

[337] Jiang H Patel PH Kohlmaier A Grenley MO McEwen DG Edgar BA. Cytokine/Jak/Stat Signaling Mediates Regeneration and Homeostasis in the *Drosophila* Midgut. Cell (2009) 137:1343–55. 10.1016/j.cell.2009.05.014 PMC2753793 19563763

[338] Nigro G Sansonetti PJ. Microbiota and Gut Stem Cells Cross-Talks: A New View of Epithelial Homeostasis. Curr Stem Cell Rep (2015) 1:48–52. 10.1007/s40778-014-0005-x

[339] Harris JM. The Presence, Nature, and Role of Gut Microflora in Aquatic Invertebrates: A Synthesis. Microb Ecol (1993) 25:195–231. 10.1007/BF00171889 24189919

[340] Hakim JA Koo H Kumar R Lefkowitz EJ Morrow CD Powell ML. The Gut Microbiome of the Sea Urchin, *Lytechinus variegatus*, From Its Natural Habitat Demonstrates Selective Attributes of Microbial Taxa and Predictive Metabolic Profiles. FEMS Microbiol Ecol (2016) 92:fiw146. 10.1093/femsec/fiw146 27368709 PMC5975844

[341] Petersen JM Osvatic J. Microbiomes in Natura: Importance of Invertebrates in Understanding the Natural Variety of Animal-Microbe Interactions. mSystems (2018) 3:2. 10.1128/msystems.00179-17 PMC5850079 29556539

[342] Faddetta T Ardizzone F Faillaci F Reina C Palazzotto E Strati F. Composition and Geographic Variation of the Bacterial Microbiota Associated With the Coelomic Fluid of the Sea Urchin. Paracentrotus lividus Sci Rep (2020) 10:1–12. 10.1038/s41598-020-78534-5 PMC7723044 33293569

[343] Schwob G Cabrol L Poulin E Orlando J. Characterization of the Gut Microbiota of the Antarctic Heart Heart Urchin (Spatangoida). Abatus agassizii. Front Microbiol (2020) 11:308. 10.3389/fmicb.2020.00308 32184772 PMC7058685

[344] Liberti A Natarajan O Atkinson CGF Sordino P Dishaw LJ. Reflections on the Use of an Invertebrate Chordate Model System for Studies of Gut Microbial Immune Interactions. Front Immunol (2021) 12:642687. 10.3389/fimmu.2021.642687 33717199 PMC7947342

[345] Yu J Peng J Chi H. Systems Immunology: Integrating Multi-Omics Data to Infer Regulatory Networks and Hidden Drivers of Immunity. Curr Opin Syst Biol (2019) 15:19–29. 10.1016/j.coisb.2019.03.003 32789283 PMC7418879

[346] Villa A Sonis ST. System Biology. In: Sonis ST Villa A, editors. Translational Systems Medicine and Oral Disease. Academic Press: London (2020). p. 9–16. 10.1016/B978-0-12-813762-8.00002-5

[347] Heintz-Buschart A May P Laczny CC Lebrun LA Bellora C Krishna A. Integrated Multi-Omics of the Human Gut Microbiome in a Case Study of Familial Type 1 Diabetes. Nat Microbiol (2016) 2:1–13. 10.1038/nmicrobiol.2016.180 27723761

[348] Thaiss CA Zmora N Levy M Elinav E. The Microbiome and Innate Immunity. Nature (2016) 535:65–74. 10.1038/nature18847 27383981

